# Speciation Studies of Bifunctional 3-Hydroxy-4-Pyridinone Ligands in the Presence of Zn^2+^ at Different Ionic Strengths and Temperatures

**DOI:** 10.3390/molecules24224084

**Published:** 2019-11-12

**Authors:** Anna Irto, Paola Cardiano, Salvatore Cataldo, Karam Chand, Rosalia Maria Cigala, Francesco Crea, Concetta De Stefano, Giuseppe Gattuso, Nicola Muratore, Alberto Pettignano, Silvio Sammartano, M. Amélia Santos

**Affiliations:** 1 Dipartimento di Scienze Chimiche, Biologiche, Farmaceutiche e Ambientali, Università di Messina, Viale F. Stagno d’Alcontres 31, 98166 Messina, Italy; airto@unime.it (A.I.); pcardiano@unime.it (P.C.); rmcigala@unime.it (R.M.C.); fcrea@unime.it (F.C.); cdestefano@unime.it (C.D.S.); ggattuso@unime.it (G.G.); 2 Dipartimento di Fisica e Chimica Emilio Segrè, ed. 17, Università di Palermo, Viale delle Scienze, I-90128 Palermo, Italy; salvatore.cataldo@unipa.it (S.C.); nicola.muratore@unipa.it (N.M.); alberto.pettignano@unipa.it (A.P.); 3 Centro de Química Estrutural, Instituto Superior Técnico, Universidade de Lisboa, Av. Rovísco Pais 1, 1049-001 Lisboa, Portugal; kc4chemistry@gmail.com

**Keywords:** 3-hydroxy-4-pyridinone, speciation, acid–base properties, extended Debye–Hückel, Zn-complexation, specific ion interaction theory, van’t Hoff equation, sequestering ability

## Abstract

The acid–base properties of two bifunctional 3-hydroxy-4-pyridinone ligands and their chelating capacity towards Zn^2+^, an essential bio-metal cation, were investigated in NaCl aqueous solutions by potentiometric, UV-Vis spectrophotometric, and ^1^H NMR spectroscopic titrations, carried out at 0.15 ≤ *I*/mol ^−1^ ≤ 1.00 and 288.15 ≤ *T*/K ≤ 310.15. A study at *I* = 0.15 mol L^−1^ and *T* = 298.15 K was also performed for other three Zn^2+^/*L*^z−^ systems, with ligands belonging to the same family of compounds. The processing of experimental data allowed the determination of protonation and stability constants, which showed accordance with the data obtained from the different analytical techniques used, and with those reported in the literature for the same class of compounds. ESI-MS spectrometric measurements provided support for the formation of the different Zn^2+^/ligand species, while computational molecular simulations allowed information to be gained on the metal–ligand coordination. The dependence on ionic strength and the temperature of equilibrium constants were investigated by means of the extended Debye–Hückel model, the classical specific ion interaction theory, and the van’t Hoff equations, respectively.

## 1. Introduction

The 3-hydroxy-4-pyridinones (3,4-HPs) are a family of compounds that are derivatives of deferiprone (DFP), which have been extensively developed as possible strong chelators and metal-related pharmaceutical drugs, due to their important roles in pharmaceutical and bioenvironmental processes, in the sequestration or release of specific metal cations (M^n+^) from or into the human body, and as metal carriers for therapeutics or imaging purposes [1,2]. These compounds feature a 6-membered *N*-heterocyclic aromatoid ring with hydroxyl and ketone groups in the ortho position, conferring them a significant binding ability towards hard divalent and trivalent metal cations (M^2+^, M^3+^) [3]. In the last two decades, their development has been considerably spreading, since several studies [1,3,4] have demonstrated that the 3-hydroxy-4-pyridinones may strongly sequester metal cations (Fe^3+^, Al^3+^, etc.), resulting in the formation of species with a higher thermodynamic stability with respect to the precursor deferiprone [5,6]. At the same time, they can be considered good alternatives to deferoxamine (DFO or Desferal^®^) [7] as iron chelators, because the use of 3,4-HPs does not involve serious drawbacks, such as high toxicity and costs, oral activity, or other possible undesired side effects. As already reported in previous papers [8,9], the 3-hydroxy-4-pyridinones can also be extra-functionalized to improve their lipophilic–hydrophilic balance and increase their affinity towards cells biological membranes. 

**Figure 1:**
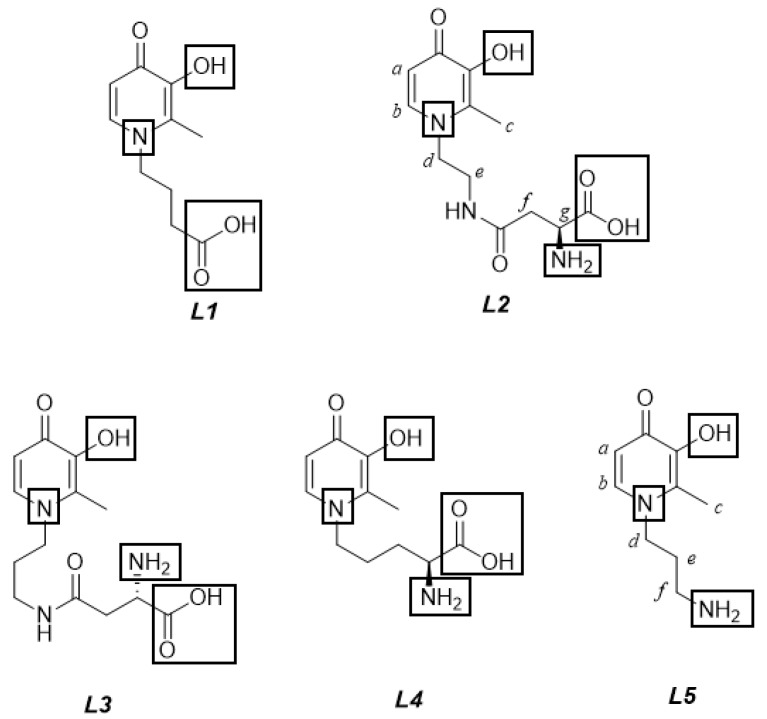
Structures of the 3-hydroxy-4-pyridinones under study, with protonable groups highlighted with rectangles. The letters represent for the proton nuclear magnetic resonance (^1^H NMR) titration peak assignment.

The present paper investigates the acid–base properties of two bifunctional 3,4-HPs, ligands (*L2*: (*S*)-2-amino-4-((2-(3-hydroxy-2-methyl-4-oxopyridin-1(4*H*)-yl)ethyl)amino)-4-oxobutanoic acid and *L5*: 1-(3-aminopropyl)-3-hydroxy-2-methylpyridin-4(1*H*)-one, Figure 1), at different ionic strength and temperature conditions (0.15 ≤ *I*/mol L^−1^ ≤ 1.00*,* 288.15 ≤ *T*/K ≤ 310.15) in NaCl_(aq)_. This ionic medium was selected because it is the principal inorganic component of many natural and biological fluids [10,11,12]. Therefore, performing studies under the experimental conditions of these fluids, namely at *I* ~ 0.01–0.10 mol L^−1^ and 0.70 mol L^−1^ for fresh and marine waters, or *I* ~ 0.16 mol L^−1^ for blood plasma, allows simulation and possibly predictions of the behaviour of real systems [13,14]. Moreover, although these 3,4-HPs metal chelators have already proven to have an inherently high affinity towards *hard* metal cations, such as Al^3+^ [8], it is interesting to study their interaction with biologically relevant M^2+^, in order to assure that along with their sequestering role of *hard* M^3+^, they do not lead to a significant depletion of important divalent bio-metal cations, in particular Zn^2+^**.** In fact, Zn^2+^ is an essential trace mineral required for the metabolism of several enzymes, cell division processes, DNA, and protein synthesis [15]. Furthermore, it has an important role in the proper growth and development of the human body, helps to protect the skin and muscles from premature aging, and has antioxidant functions against free radicals [16]. Zinc also participates in regulation of immune functions through the activation of T lymphocytes (T cells), which help the body to control and regulate the immune responses and to attack infected or cancerous cells [17]. Therefore, the binding ability of the cited 3,4-HPs towards Zn^2+^ was investigated, as well as for three other ligands (*L1*: 4-(3-hydroxy-2-methyl-4-oxopyridin-1(4*H*)-yl)butanoic acid, *L3*: (*S*)-2-amino-4-((3-(3-hydroxy-2-methyl-4-oxopyridin-1(4*H*)-yl)propyl)amino)-4-oxobutanoic acid, and *L4:* (S)-2-amino-5-(3-hydroxy-2-methyl-4-oxopyridin-1(4*H*)-yl)pentanoic acid; Figure 1), all of which belong to the same class of compounds. From an experimental point of view, potentiometric measurements, using specific electrodes for H^+^ ion activity (ISE-H^+^), and ultraviolet-visible (UV-Vis) spectrophotometric measurements were carried out at *I* = 0.15 mol L^−1^ in NaCl_(aq)_ and *T* = 298.15 K, and in the case of *L2* and *L5* 3-hydroxy-4-pyridinones, also at 0.15 ≤ *I*/mol L^−1^ ≤ 1.00*,* 288.15 ≤ *T*/K ≤ 310.15. Electrospray mass (ESI-MS) spectrometric measurements were performed to investigate the possible formation of Zn^2+^/ligand species with different stoichiometry at *I* = 0.15, 1.00 mol L^−1^ in NaCl_(aq)_ and in the absence of ionic medium, at *T* = 298.15 K. Proton nuclear magnetic resonance (^1^H NMR) spectroscopic titrations and computational studies were also carried out to gain information on the Zn^2+^–ligand coordination mode. The protonation and stability data, determined for the different experimental conditions, were used to model the dependence of thermodynamic parameters on ionic strength by means of the extended Debye–Hückel (EDH) equation and the specific ion interaction theory (SIT), while the effect of temperature was determined using the van’t Hoff equation. Finally, the sequestering ability of the 3-hydroxy-4-pyridinones towards the metal cation under study was investigated by calculating the empirical parameter pL_0.5_, already proposed in [18], at different pH, ionic strength, and temperature conditions. 

## 2. Results and Discussion

### 2.1. Equilibria and Thermodynamic Models

The acid–base properties of the ligands (*L*^z−^) were investigated, taking into account the following stepwise (log*K*_r_^H^; Equation (1)) and overall (log*β*_r_^H^; Equation (2)) equilibria, respectively:

H^+^ + H_(r − 1)_*L*^−(z−(r − 1))^ = H_r_*L*^−(z − r)^     log*K*_r_^H^ (1)

and

rH^+^ +*L*^z−^ = H_r_*L*^−(z−r)^       log*β*_r_^H^ (2)

where r is the r^th^ protonation step; z is the charge of the completely deprotonated 3-hydroxy-4-pyridinones.

The metal hydrolytic behavior is described by the equilibrium:

pZn^2+^ + rH_2_O = Zn_p_(OH)_r_^(2p−r)^ + rH^+^      log*β*_r_^OH^ (3)

The stepwise and overall stability constants of the Zn^2+^-ligands species are given as follows:

pZn^2+^ + H_r_*L*_q_^−(zq−(r−1)^ = Zn_p_*L*_q_H_r_^(2p+r−qz)^ + H^+^     log*K*_pqr_ (4)

pZn^2+^ + q*L*^z−^ + rH^+^ = Zn_p_*L*_q_H_r_^(2p+r−qz)^      log*β*_pqr_ (5)

If ternary metal–ligand hydrolytic species are formed, equilibria refer to:

pZn^2+^ + q*L*^z−^ + rH_2_O = Zn_p_*L*_q_(OH)_r_^(2p−r−zq)^ + rH^+^    log*β*_pq-r_ (6)

The protonation and stability of formation constants, concentrations, and ionic strengths are expressed in the molar (*c*, mol L^−1^) or molal (*m*, mol (kg H_2_O)^−1^) scales. Molar to molal conversions were performed by means of appropriate density values. 

The dependence on ionic strength (*I*) of the thermodynamic parameters was studied using an extended Debye–Hückel-type equation [19]:

log*K* = log*K*^0^ − z*·DH + C·*I* (7)

where log*K*^0^ is the equilibrium constant atinfinite dilution; z* is the Σ (charge)^2^_reactants_ − Σ (charge)^2^_products_; DH is the 0.51·(*I*^1/2^/(1 + 1.5*I*^1/2^)), Debye–Hückel term; C is the empirical parameter for the dependence of the equilibrium constants on the ionic strength. 

If the equilibrium constants are expressed in the molal concentration scale, Equation (7) can be modified to obtain the classical specific ion interaction theory (SIT) [20,21,22], where C parameter is replaced by Δε:

Δε_ij_ = ∑_i_ ε(i, j) (8)

where ε(i, j) is the interaction coefficient for an i^th^ species with a j^th^ component of opposite charge.

Furthermore, protonation and formation constants determined at different temperatures were used to calculate the enthalpy values by applying the van’t Hoff equation, assuming that the contribution of ΔC_p_ ina small temperature range, in our case 288.15–310.15 K, is negligible:

log*K*_T_ = log*K*_θ_ + (Δ*H*_θ_/2.303R) (1/θ − 1/*T*) (9)

where θ is the reference temperature (298.15 K); *T* is temperature in Kelvin; Δ*H*_θ_ is enthalpy change at reference temperature; R is 8.314 J K^−1^ mol^−1^ and is the universal gas constant.

To complete the thermodynamic picture of the Zn^2+^/ligand system behavior in NaCl aqueous solution, the Gibbs free energy was calculated from the equilibrium constants, as reported by Equation (10):

Δ*G* = −R*T* ln*K* (10)

From the knowledge of the enthalpy changes and the Gibbs free energy, the *T*∆S values, as known, were detemined at the same experimental conditions:

*T*Δ*S* = ΔH − Δ*G* (11)

### 2.2. Protonation Constants of the Ligands

The protonation constants of the ligands (Figure 1) have already been published at *I* = 0.15 mol L^−1^ in NaCl_(aq)_ and *T* = 298.15 K and 310.15 K (Table S1) [8]. As a continuation of this study, the research group undertook a complete investigation on the acid–base properties of two 3-hydroxy-4-pyridinones, namely *L2* and *L5*, in NaCl_(aq)_ at 0.15 ≤ *I*/mol L^−1^ ≤ 1.00 and 288.15 ≤ *T*/K ≤ 310.15.

Both the ligands were synthesized in the H_r_(L)^0^ neutral species form; the possible protonable sites, as shown in Figure 1, can be assigned to:

1. the hydroxyl group of the aromatoid ring;
2. the –NH_2_ and –COOH groups, potentially present in the alkyl moiety;
3. the pyridinone nitrogen in the N-heterocyclic ring, with the proton supplied by excess of inorganic acid [8].

#### 2.2.1. *L2* Behavior in Aqueous Solution

*L2* ligand is an aspartic acid 3-hydroxy-4-pyridinone hybrid featuring all of the abovementioned protonable groups; its acid–base properties were investigated by means of UV-Vis spectrophotometric measurements, carried out at different ionic strengths and temperatures in NaCl aqueous solution.

Analysis of the experimental data led, in all cases, to the determination of four protonation constants, namely log*K*_1_^H^ (9.88–10.99), log*K*_2_^H^ (6.03–9.32), log*K*_3_^H^ (3.97–4.93), and log*K*_4_^H^ (3.06–3.73), as reported in Table 1 and Table S2 (in molar and molal concentration scales, respectively). These were already attributed, by means of ^1^H NMR titrations, to –OH, –NH_2_, –COOH, and the pyridinone nitrogen atom, respectively. Table S3 shows the average chemical shift values calculated for each protonated species at *I* = 0.15 mol L^−1^ in NaCl_(aq)_ and *T* = 298.15 K [8]. 

**Figure 2:**
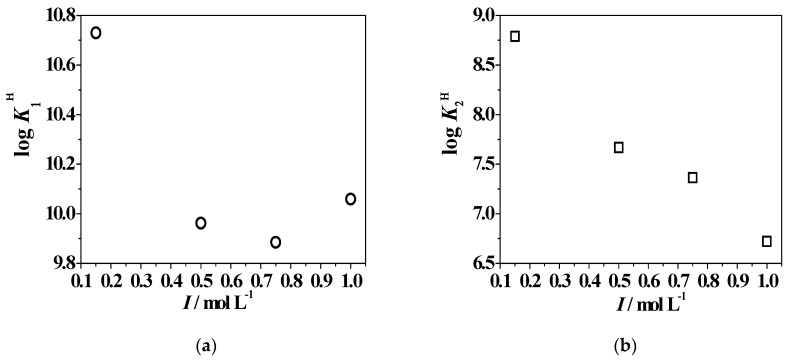

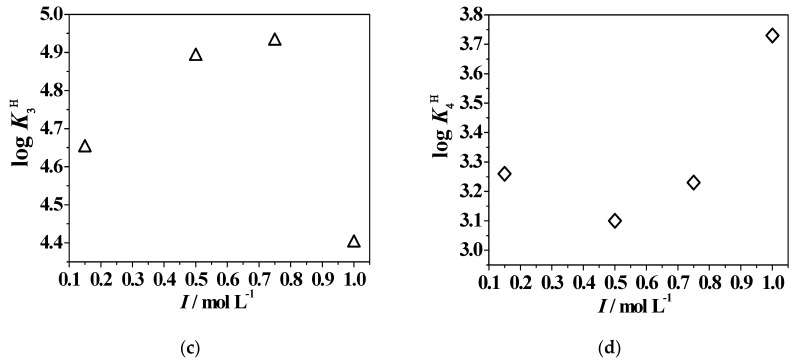
Trend of log*K*_1_^H^ (**a**), log*K*_2_^H^ (**b**), log*K*_3_^H^ (**c**), and log*K*_4_^H^ (**d**) *L2* protonation constants vs. the ionic strength (in mol L^−1^) in NaCl_(aq)_ and *T* = 298.15 K.

As examples of the ionic strength effect on the acid–base properties of the ligand, the protonation constant trends vs. *I* are shown in Figure 2, clearly indicating that the behavior of each protonable site is different than the others. With the exception of –NH_2_ group, whose log*K*_2_^H^ values constantly decrease with increasing ionic strength, the remaining sites undergo a tendency inversion at *I* = 0.75–1.00 mol L^−1^ in NaCl_(aq)_.

**Figure 3:**
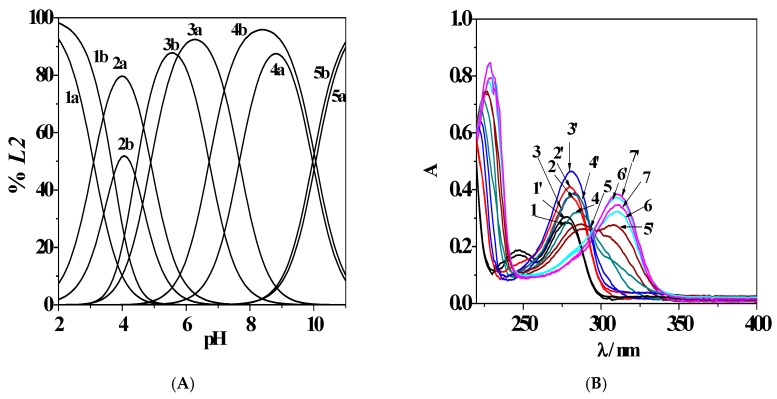
(**A**) Distribution diagram of *L2* (*c_L_* = 5.3·10^−5^ mol L^−1^) species at *T* = 298.15 K and *I* = 0.506 (a) and 1.012 (b) mol L^−1^ in NaCl_(aq)_. Species: (1) H_4_(*L2*)^2+^; (2) H_3_(*L2*)^+^; (3) H_2_(*L2*)^0^_(aq)_; (4) H(*L2*)^−^; (5) (*L2*)^2−^. (**B**) UV-Vis spectrophotometric titration curves of *L2* underthe same experimental conditions as for the distribution diagram and at different pH values. At *I* = 0.506 mol L^−1^: (1) pH = 2.01, λ_max_ = 278 nm; (2) pH = 3.62, λ_max_ = 275 nm; (3) pH = 4.90, λ_max_ = 283 nm; (4) pH = 7.39, λ_max_ = 285 nm; (5) pH = 9.68, λ_max_ = 290 nm; (6) pH = 10.56, λ_max_ = 310 nm; (7) pH = 11.00, λ_max_ = 310 nm. At *I* = 1.012 mol L^−1^: (1′) pH = 2.00, λ_max_ = 278 nm; (2′) pH = 3.59, λ_max_ = 279 nm; (3′) pH = 4.50, λ_max_ = 280 nm; (4′) pH = 8.40, λ_max_ = 283 nm; (5′) pH = 9.84, λ_max_ = 296 nm; (6′) pH = 10.60, λ_max_ = 310 nm; (7′) pH = 10.99, λ_max_ = 311 nm.

Furthermore, a comparison between protonated species distribution at *T* = 298.15 K*, I* = 0.506 mol L^−1^, and 1.012 mol L^−1^ is reported in Figure 3A. The H_4_(*L2*)^2+^ species is present at pH ~ 2.0, with percentages higher than 90% at both experimental conditions, while the H_3_(*L2*)^+^ reaches the 80% and 51% of formation at pH ~ 4.0 with increasing ionic strength. The bis-protonated H_2_(*L2*)^0^_(aq)_ species is characterized by an opposite trend, achieving its maximum (88%) at lower pH (pH ~ 5.5) and with *I* = 1.012 mol L^−1^ than with *I* = 0.506 mol L^−1^ (pH ~ 6.3, 93%). The H(*L2*)^−^ starts to form at pH ~ 4.2 and reaches 87% and 96% of formation at pH ~ 8.9 and 8.4, with the variable increasing. At last, the completely deprotonated species (*L2*)^2−^ reaches about 90% at both ionic strength conditions. The effect of this variable can be further examined by the analysis of Figure 3B, where a comparison between the UV-Vis titration curves is reported, recorded at the same experimental conditions as those used to draw the distribution diagram. 

The absorbance spectra vary with pH increase due to the different behaviours of the protonated species. In all cases, as already found at *I* = 0.15 mol L^−1^ in NaCl_(aq)_ and *T* = 298.15 K [8], an absorption band at λ_max_ = 278 nm and pH ~ 2.0 was recorded, featuring an increase of intensity at pH ~ 4.5 and 4.9 at *I* = 0.506 mol L^−1^ and 1.012 mol L^−1^, respectively. A batochromic shift and the formation of different isosbestic points were noticed. Furthermore, a general intensity increase of each UV-Vis absorbance maximum with increasing ionic strength was observed across the pH range investigated, probably due to a noteworthy contribution of the ionic medium to the ligand speciation in aqueous solution.

The deconvolution of the UV-Vis spectrophotometric data allowed us to calculate the molar absorbivities (ε/mol^−1^ L cm^−1^) of each species; as an example, the ε variation with pH is reported in Figure S1 at *T* = 298.15 K and *I* = 0.506 mol L^−1^ and 1.012 mol L^−1^ in NaCl_(aq)_. The values of the molar absorbivities in these conditions are:

- ε_max_(H_4_(*L2*)^2+^) = 5221 and 5742 at λ_max_ = 278 nm with increasing ionic increasing;
- ε_max_(H_3_(*L2*)^+^) = 8756 and 9400 at λ_max_ = 291 nm, *I* = 0.506 mol L^−1^ and 1.012 mol L^−1^, respectively;
- ε_max_(H_2_(*L2*)^0^_(aq)_) = 7468 and 9681 at λ_max_ = 284 nm and 281 nm, respectively;
- ε_max_(H(*L2*)^−^) = 5016 at λ_max_ = 296 nm and both the variable conditions;
- ε_max_((*L2*)^2−^) = 7913 and 8276 at λ_max_ = 311 nm, with increasing ionic strength.

To investigate the effect of temperature on the speciation of the ligands in NaCl aqueous solution, UV-Vis spectrophotometric measurements were performed at *I* = 0.15 mol L^−1^ and *T* = 288.15 K, which together with the data already published in the literature at *T* = 298.15 K and 310.15 K [8] showed a trend for protonation constants with the considered variables. Analyzing the data reported in Table 1 and Table S1 (in molar and molal concentration scales, respectively), a different tendency was found for the hydroxyl group than for the others (carboxylic, amino, and pyridinone nitrogen groups); log*K*_1_^H^ values increased with temperature, while for the other protonation constants an opposite trend can be observed. In particular, the significant variation of log*K*_2_^H^ for *T* = 288.15 K–298.15 K and *T* = 310.15 K could probably be explained by the literature data, which reported a compound similar to *L2*, mimosine. It was observed that the contribution of the ligand structure to the resonance hybrid leading to a partial positive charge on the pyridinone nitrogen ring [23], together with the possible temperature effect, could cause a noteworthy decrease of protonation constants, attributed to the –NH_2_ group, in comparison with the typical values usually obtained for this protonable site (log*K*^H^ ~ 9.0–9.5) [24].

In Figure S2, a comparison between the distribution diagrams of the ligand at *I* = 0.15 mol L^−1^ in NaCl_(aq)_, *T* = 288.15 K, and *T* = 310.15 K showed that the species formation percentages decrease with temperature increase. In physiological conditions, they are shifted towards lower pH values, with the exception of the H(*L2*)^−^ species, which, as already evidenced, displays different behavior than the other ones compared with the considered variable. 

#### 2.2.2. *L5* Ligand Protonation

The second 3-hydroxy-4-pyridinone under study, *L5*, shown in Figure 1, features three protonable moieties, namely the hydroxyl group, the pyridinone nitrogen atom, and the terminal amine group of the alkylic chain. 

^1^H NMR measurements were carried out at *I* = 0.15 mol L^−1^ in NaCl_(aq)_ and *T* = 298.15 K to further analyze *L5* acid–base behavior, which was previously investigated using UV-Vis spectrophotometric and spectrofluorimetric techniques [8]. The protonation constants (Table 1), refined using the HypNMR computer program, were in good agreement with those previously reported [8]. The collected spectra show a single set of signals shifted upfield with pH increase. As expected, all the resonances of protons closer to the pyridinone nitrogen of the aromatoid ring are more or less shielded in the pH range 2–4.5, thus confirming that this is the first group to be deprotonated, as already observed in similar ligands [8]. In detail, the *a* and *b* signals are characterized by a comparable upfield shift (0.48 and 0.33 ppm, respectively) in the cited pH range, whereas they do not change to a great extent from pH 4.5 to 7.5 and start to decrease again at alkaline pH. A less significant upfield shift can also be observed for *c* and *d* protons at pH < 4.5, and once again, the chemical shifts follow the behavior already discussed for the pyridinone protons. The other two signals, namely *e* and *f*, show an opposite trend with pH increase, since they start to considerably change from neutral pH and greater, confirming the deprotonation sequence already reported.

From the speciation data it is known that at pH ~ 2.0 the H_3_(*L5*)^2+^ species should be present in solution with a percentage of ca. 95%, whilst between 5.2 and 6.4 the most abundant species should be the H_2_(*L5*)^+^*,* reaching 99.8%; the spectra recorded in these conditions were compared with the calculated chemical shifts obtained by HypNMR for the same species. The calculated δ values for H_3_(*L5*)^2+^ and H_2_(*L5*)^+^, listed in Table S3, are in excellent agreement with the ones observed in the spectra recorded at the pH level where these species reach the maxima. In addition, Figure S3 shows the almost total overlap between observed and calculated chemical shifts for some selected nuclei. 

The acid–base properties of *L5* were also studied by potentiometric measurements performed under different ionic strength and temperature conditions in NaCl_(aq)_. The treatment of the experimental data allowed the determination of three protonation constants (Table 1 and Table S1): log*K*_1_^H^ (9.79–11.20), log*K*_2_^H^ (5.96–6.92), and log*K*_3_^H^ (3.00–4.06). 

**Figure 4:**
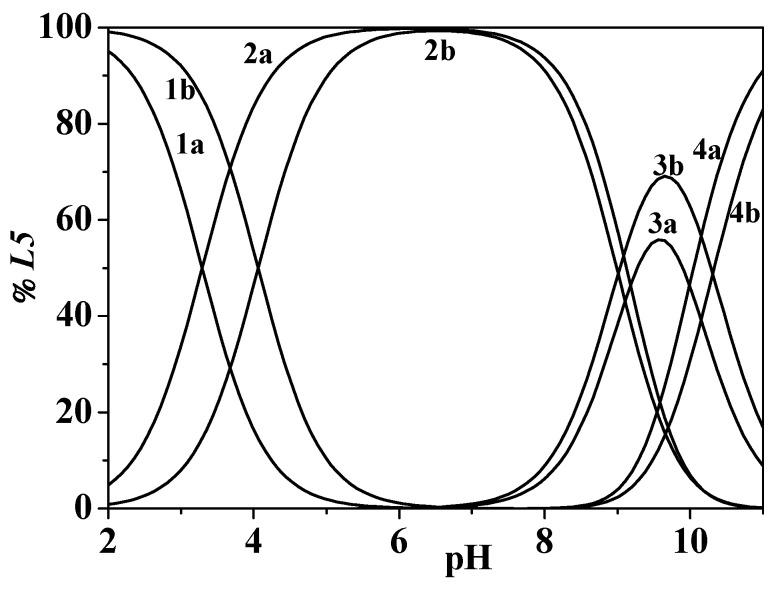
Distribution diagram of *L5* (*c*_L_ = 1.0·10^−3^ mol L^−1^) species at *T* = 298.15 K, *I* = 0.473 (a) and *I* = 1.008 (b) mol L^−1^ in NaCl_(aq)_. Species: (1) H_3_(*L5*)^2+^; (2) H_2_(*L5*)^+^; (3) H(*L5*)^0^_(aq)_; (4) (*L5*)^−^.

The effect of the ionic strength on the protonation of the *L5* ligand can be observed in Figure 4 and Figure S4. In the first case, the trend of log*K*_r_^H^ vs. *I* is reported, showing for this ligand a behavior similar to that found for analogous functional groups in *L2*. Therefore, the absence of the amidic moiety or the carboxyl group in the *L5* ligand structure, with respect to the first ligand, would not seem to influence the effect of theionic strength increasing on the acid–base properties of the 3-hydroxy-4-pyridinone.

In Figure 4, the ligand speciation diagrams, drawn at *T* = 298.15 K, *I* = 0.473 mol L^−1^, and *I* = 1.008 mol L^−1^ in NaCl_(aq)_, show that all the species are uniformly distributed along the pH range investigated. The H_3_(*L5*)^2+^ species is present in solution at pH ~ 2.0, reaching more than 90% in both the experimental conditions. This species achieves the maximum formation percentage at pH ~ 6.4; at pH ~ 9.6, H(*L5*)^0^_(aq)_ reaches 56% and 70% and (*L5*)^−^ reaches 91% and 83% at *I* = 0.473 mol L^−1^ and *I* = 1.008 mol L^−1^, respectively. Therefore, this figure shows a trend of protonations occurring at slightly higher pH values with ionic strength increasing.

Regarding the temperature effect, potentiometric data determined at *I* = 0.15 mol L^−1^ in NaCl_(aq)_ and *T* = 288.15 K allowed us to obtain a trend for protonation constants, together with the values already determined at *T* = 298.15 K and in physiological conditions [8]. Table 1 and Table S1 show that log*K*_1_^H^ values increase from the lowest temperature to *T* = 298.15 K, followed by an inversion of tendency at *T* = 310.15 K. For log*K*_2_^H^, the variation of some logarithmic units on the protonation constant at physiological temperature was already discussed in the previous subparagraph (*L2* behavior in aqueous solution) [23]. Log*K*_3_^H^, attributed to the pyridinone nitrogen atom, similar to the same moiety in *L2*, is characterized by a decrease with variable increase. To better observe the temperature effect on the speciation of the ligand in NaCl aqueous solution, in Figure S5 a comparison between the distribution diagrams of *L5* at *I* = 0.15 mol L^−1^ in NaCl_(aq)_, *T* = 288.15 K, and *T* = 310.15 K is depicted. The figure clearly indicates that the formation of protonated species occurs at higher pH values with temperature increasing.

### 2.3. Hydrolysis of the Metal Cation

The acid–base properties of Zn^2+^ in NaCl_(aq)_ were already known and have been reported in the literature under different ionic strength and temperature conditions (Table S4) [6,25,26].

### 2.4. Binding Ability Towards Zn^2+^

The investigation on the Zn^2+^ interactions with the bifunctional 3-hydroxy-4-pyridinones (*L1*–*L5*) led to the determination of species with different stoichiometry (Zn_p_*L*_q_H_r_^(2p+r−qz)^). The best possible speciation schemes were selected considering different criteria, such as:

- simplicity and probability of the model;
- formation percentages of the species across the pH range under investigation;
- statistical parameters (standard deviation on log*β*_pqr_ values and on the fitting values of the systems);
- values of corresponding ratios with single variances in comparison with those from the accepted model.

The high number of experiments performed (and of experimental points collected) showed the differences in variance between the accepted model and other models to be significant. 

In Table 2, the stability constants of all of the Zn^2+^/3-hydroxy-4-pyridinones complex species are reported at *I* = 0.15 mol L^−1^ in NaCl_(aq)_ and *T* = 298.15 K. The experimental data, whenever possible, were determined by means of potentiometry (1^st^ column), UV–Vis spectrophotometry (2^nd^ column), and ^1^H NMR spectroscopy (3^rd^ column); the average (4^th^ column) of the obtained values was also calculated. The measurements were carried out in the pH ranges of 2.0–10.5 for potentiometric and UV–Vis investigations, and 2.0–8.1 for ^1^H NMR ones. 

As can be inferred from the analysis in Table 2, for the mentioned experimental conditions, a trend of stability of the species can be observed due to a common complex, namely Zn*L*^(2−z)^:

*L4* > *L3* > *L5* > *L2* > *L1*

This trend could be explained by assuming that the stability of the Zn^2+^/*L*^z−^ systems may be favored by the simultaneous presence on the ligand molecules of carboxylic and amino groups or even different alkyl chains; in fact, there is a decrease with decreasing alkyl chain length (*L2*) and with the absence of an amino group (*L1*). In the case of *L2* and *L5*, experiments at 0.15 ≤ *I*/mol L^−1^ ≤ 1.00 and 288.15 ≤ *T*/(K≤ 310.15 were also carried out to give a more complete thermodynamic picture of the Zn^2+^/ligand systems.

#### 2.4.1. Zn^2+^/*L1*, *L3* and *L4* Systems

**Figure 5:**
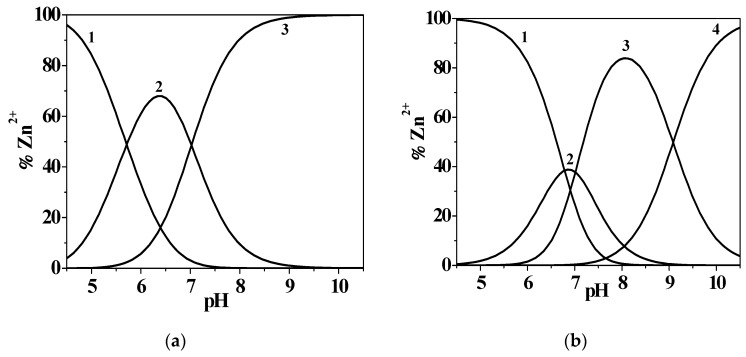
Distribution diagrams of Zn^2+^/*L1* (**a**) and *L4* (**b**) (*c_Zn_^2+^* = 4.310^−4^ mol L^−1^, *c_L_* = 1.2·10^−3^ mol L^−1^) species at *I* = 0.150 mol L^−1^ in NaCl_(aq)_, *T* = 298.15 K. (**a**) Species: (1) free Zn^2+^; 2. Zn(*L1*)^0^_(aq)_; (3) Zn(*L1*)OH^−^. (**b**) Species: (1) free Zn^2+^; 2. Zn(*L4*)H^+^; (3) Zn(*L4*)^0^_(aq)_; (4) Zn(*L4*)OH^−^.

The study on Zn^2+^/*L1* complexation allowed the determination of a model characterized by two species: Zn(*L1*)^0^_(aq)_ and Zn(*L1*)OH^−^. The distribution diagram reported in Figure 5a, drawn at *I* = 0.146 mol L^−1^ in NaCl_(aq)_ and *T* = 298.15 K, shows that the metal–ligand interaction starts at pH ~ 3.9 with the formation of Zn(*L1*)^0^_(aq)_ speciesreaching the 68% of formation at pH ~ 6.3, while the mixed hydroxo complex achieves its maximum formation percentage at pH ~ 10.5. 

*L3* and *L4* ligands, as shown in Figure 1, feature another protonable site, namely the –NH_2_ group, with respect to *L1*; *L3* is also characterized by an amidic moiety close to the amino and carboxylic groups.

The speciation schemes obtained for these Zn^2+^/3,4-HPs systems are characterized by the formation of Zn*L*^0^_(aq)_, Zn*L*OH^−^, and Zn*L*H^+^ species. As listed in Table 2, the stability constants determined for Zn^2+^/*L3* and *L5* ligands are higher than the ones obtained for *L1*, indicating that a possible involvement of the –NH_2_ and amidic moieties could not be excluded in the metal–ligand interaction (for further details, see Section 2.4.2. Zn^2+^/*L2* system section); furthermore, in the case of the Zn^2+^/*L4* system, the formationconstant values at *I* = 0.15 mol L^−1^ in NaCl_(aq)_ and *T* = 298.15 K are in good agreement among the different analytical techniques used. Figure 5b shows a distribution diagram drawn at *I* = 0.147 mol L^−1^ in NaCl_(aq)_ and *T* = 298.15 K for the Zn^2+^/*L4* system. 

With respect to the previous diagram, in this case the metal–ligand complexation starts at a higher pH value (pH ~ 4.7), in correspondence with the Zn(*L4*)H^+^ species, which reaches 39% formation at pH ~ 6.9, while the simple 1:1 stoichiometry and the mixed-hydroxo complexes achieve 84% and 95% formation at pH ~ 8.0 and 10.5, respectively. The Zn^2+^/*L3* system displays a similar distribution, as observable in Figure S6.

#### 2.4.2. Zn^2+^/*L2* Investigation

The solution study of the *L2* ligand in the presence of Zn^2+^ was performed by means of potentiometric, UV-Vis spectrophotometric, and ^1^H NMR investigation at *I* = 0.15 mol L^−1^ in NaCl_(aq)_ and *T* = 298.15 K. The determined speciation model is characterized by three species, namely Zn(*L2*)H^+^, Zn(*L2*)^0^_(aq)_, and Zn(*L2*)OH^−^. In Table 2, the stability constants, determined with quite good accordance among the different analytical techniques, are reported together with the average of the obtained results. These last data are called “suggested values”, which are useful for describing the system behavior in a more complete way by taking into account different component concentrations, namely high concentrations (*c*_L_ ~ 10^−2^–10^−3^ mol L^−1^) in potentiometric and ^1^H NMR measurements, and low concentrations (*c*_L_ ~10^−5^ mol L^−1^) in UV-Vis spectrophotometric measurements.

**Figure 6:**
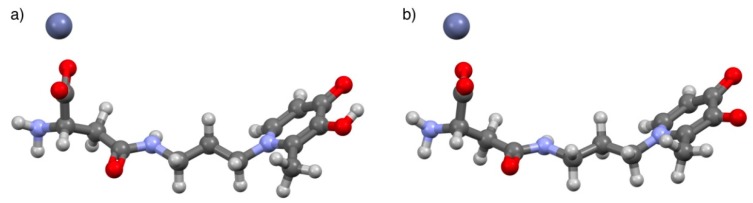
Ball-and-stick views of the PM3-optimized geometry (calculated within a cluster of 100 explicit water molecules) of the Zn^2+^/*L2* species: (**a**) Zn(*L2*)H^+^ and (**b**) Zn(*L2*)^0^_(aq)_. Oxygen = red; carbon = grey; hydrogen = white, nitrogen = blue; zinc = dark blue.

The acid–base properties of *L2* have already been studied by ^1^H NMR spectroscopy and reported elsewhere [8]; conversely, the titrations performed in the presence of Zn^2+^ are herein commented on for the first time. The measurements were stopped at about pH ~ 8.1 due to the formation of sparingly soluble species. All the collected spectra showed only a single set of peaks, thus proving that the species formed in the solution present fast exchange on the NMR time scale, as observed for similar systems [4,27]. From the comparison of the data collected from the Zn^2+^/*L2* system with that of free *L2*, it can be argued that the presence of the metal cation leaves the signals referred to as *a*, *b*, *c*, and *d* almost unchanged below pH 4.5–5 (i.e., the protons closer to the hydroxo-oxo moiety). In detail, *a*, *c*, and *d* peaks show a common behavior, being slightly deshielded with respect to the corresponding signals of the free ligand, starting from approximately pH 5.0. At the same time, the resonance due to *b* undergoes a downfield shift compared to the *L2* system from pH 4.5 onwards. The non-equivalent *f* protons, here reported as *f*_1_ and *f*_2_, display an opposite trend due to the presence of Zn^2+^, with one being shielded and the other deshielded, starting from a more acidic pH than before (i.e., approximately 3.2). The *e* methylene as well as *g* methyne protons seem to be unaffected by the presence of the metal along the investigated pH range. It is worth remembering that each observed shift resulting from fast-exchanging species corresponds to an averaged shift, so that apparently some signals may not change upon pH increase or in the presence of metal as a result of mutual exchange. In this case, the calculation of the single resonances for each nucleus of the species present in equilibrium is required according to the selected speciation model, to gain deeper insight into the system in solution. Accordingly, by comparing the chemical shifts of the expected species calculated by means of HypNMR (Table S5) with the free ligand ones, it appears that for Zn(*L2*)H^+^ the peaks more affected by the presence of the metal cation are *a*, *b*, *f*_1_, *f*_2_, and *g*, suggesting that the interactions occur both *via* hydroxo-oxo as well as amide part of the ligand. In the case of the other expected species, namely Zn(*L2*)^0^_(aq)_, the shifts, although less pronounced, involve the same protons, so that similar conclusions may be drawn. On these bases, it appears that for both species all the coordination sites of *L2* allow the formation of the complexes, even though we do not feel confident enough to claim that clear indications have been obtained from NMR investigations for this system. Regardless, once again NMR studies provide useful information about the reliability of the system speciation profile. Further evidence of the structural features of the complexes was gathered by computer modeling. Preliminary attempts to calculate the preferred conformation of Zn(*L2*)H^+^ and Zn(*L2*)^0^_(aq)_ in vacuo at the functional density level of theory did not produce reliable minima. It was, therefore, envisaged that placement of the ligand *L2* and the Zn^2+^ cation within a cluster of 100 explicit water molecules would provide a more accurate description of the zinc–ligand interaction [28]. PM6 (Parameterization Method 6) semiempirical calculations yielded minimum energy geometries for the Zn(*L2*)H^+^ and Zn(*L2*)^0^_(aq)_ complexes, both featuring the zinc cation in close proximity to the carboxylate moiety (Figure 6). Interestingly, the pyridinone oxygen atoms are not involved in complexation, regardless of their protonation state, although they are strongly solvated (water molecules not shown). Likewise, the amide moiety is seensolvated, but the zinc cation is too far away to interact with the carbonyl oxygen. The involvement of the carboxylate group is in line with the data obtained by ^1^H NMR, with special reference to the diastereotopic hydrogen atoms *f*_1_ and *f*_2_. The increased chemical shift difference between the two resonances upon the addition of Zn^2+^ may be the result of the hampered rotational freedom of the aminoacidic moiety due to the interaction of the carboxylate with the metal. In agreement with this, the methyne proton *g* also undergoes a significant shift, and the modest shift of the *e* protons confirms the passive role played by the amide group.

UV-Vis spectrophotometric titrations were also carried out at 0.15 ≤ *I*/mol L^−1^ ≤ 1.00 and 288.15 ≤ *T*/K ≤ 310.15 to evaluate the effect of ionic strength and temperature on the speciation. The model obtained at *I* = 0.15 mol L^−1^ in NaCl_(aq)_ and *T* = 298.15 K was confirmed atthe other experimental conditions and the Zn^2+^/ligand complex formation constants are reported in Table 3. 

**Figure 7:**
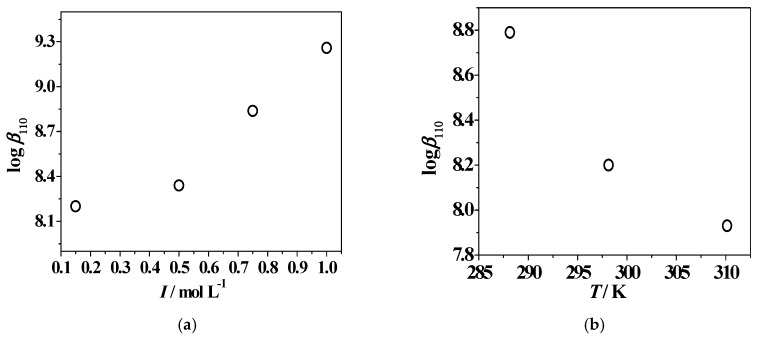
Trends of Zn^2+^/*L2* suggested log*β*_110_ values vs. the ionic strength (mol L^−1^) in NaCl_(aq)_ and *T* = 298.15 K (**a**) and vs. the temperature at *I* = 0.15 mol L^−1^ (**b**)_._

The stepwise formation constants of Zn(*L2*)H^+^ and Zn(*L2*)^0^_(aq)_ increase with *I* and decrease with *T*, as observable in Figure 7 for the metal–ligand 1:1 stoichiometry species. Concerning the hydrolytic mixed species, the stability decreases with ionic strength, but with an inversion of tendency at *I* = 0.75–1.00 mol L^−1^, whilst the effect of temperature is opposite to that of the other two species. The influence of ionic strength on the speciation can be further examined by the analysis of Figure S7, in which a comparison between the UV-Vis titration curves is reported, recorded at *I* = 0.501 mol L^−1^ and 1.005 mol L^−1^ and *T* = 298.15 K. Analogous to the spectra reported in Figure 3B, for both ionic strength conditions, the intensity of the usual band at λ_max_ = 278 nm increases up to pH ~ 4.1, as already observed for the protonation reaction. Above pH ~ 5.2, a decrease of absorbance with a bathochromic shift and a further raise of signal with respect to the protonated species occur. An increase of the UV-Vis titration curve intensity with the variable increase was also noticed for the complexes along all pH ranges investigated. 

In Figure S8a, the distribution diagram of Zn^2+^/*L2* species, drawn at the same experimental conditions employed for the UV-Vis titration curves described previously, shows that all the species reach percentages higher than 20%, and in particular, at *I* = 1.005 mol L^−1^ the formation of Zn(*L2*)H^+^ and Zn(*L2*)^0^_(aq)_ is shifted towards more acidic pH values than at *I* = 0.501 mol L^−1^. In Figure S8b, the effect of temperature on the speciation is highlighted:the beginning of metal complexation is favored at higher temperature, starting from pH ~3.5 and 5.0 at *T* = 310.15 K and 288.15 K, respectively. The Zn(*L2*)H^+^ and Zn(*L2*)OH^−^ complex formations reach higher percentages at high temperature, while Zn(*L2*)^0^_(aq)_ is characterized by an opposite trend. Low (<5%) formation percentages of the hydrolytic Zn(OH)_2_^0^_(aq)_ species are observed in both *T* conditions; at *T* = 310.15 K, these start to form at pH ~ 6.8, whereas at lower temperature values this occurs at pH > 9.0.

#### 2.4.3. Zn^2+^/*L5* System

The same analytical techniques employed for the previous system were used for the investigation of Zn^2+^/*L5* complexation at *I* = 0.15 mol L^−1^ in NaCl_(aq)_ and *T* = 298.15 K. The speciation model, considered the best possible match with the already cited criteria, is characterized by two complex species, namely Zn(*L5*)H^2+^ and Zn(*L5*)^+^. The stability constants are in good agreement among the different techniques, and the suggested values are reported in Table 2. 

**Figure 8:**
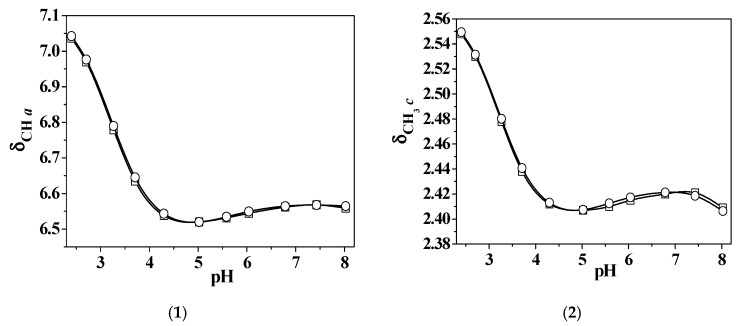

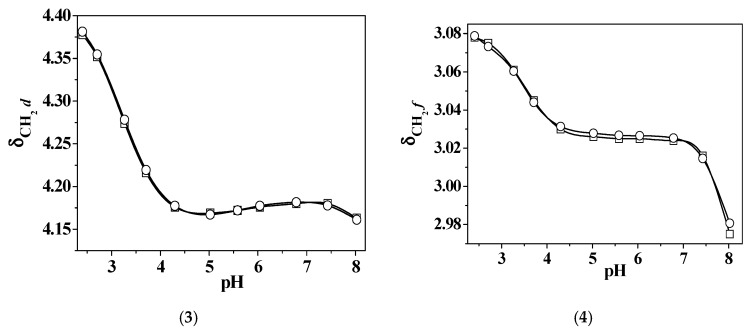
Observed (□) and calculated (○) values of chemical shifts of: *a* (**1**), *c* (**2**), *d* (**3**), and *f* (**4**) nuclei of *L5* in Zn^2+^/*L5* system vs. pH at *c*_Zn2+_ = 3.3·10^−2^ mol L^−1^, *c*_L_ = 1.0·10^−2^ mol L^−1^, and *I* = 0.15 mol L^−1^ in NaCl_(aq)_ and *T* = 298.15 K.

All ^1^H NMR investigations carried out in Zn^2+^/*L5*-containing solutions by varying the metal/ligand ratio andthe relative concentrations, show comparable spectra in the same pH conditions, as well as a single set of resonances, indicating that although complex species may be present in solution, all of them are involved in a fast mutual exchange. By comparing the spectra collected for *L5* acid–base properties with the ones recorded for the titrations on the Zn^2+^-containing solutions, it appears that up to about pH 5.0 the chemical shifts due to the protons indicated as *a*, *b*, *c*, and *d* (i.e., the ones closer to pyridinone nitrogen on the aromatoid part of *L5*), are not influenced by the presence of the metal in the solution. Conversely, upon pH increasing, *a* and *b* signal results, as well as to a lesser extent *c* and *d*, shifted with respect to the corresponding free ligand peaks. In addition, the presence of the metal in the solution does not result in any shifting of *e* and *f* signals up to pH ~8.0; it is worth mentioning that the titrations stopped at approximately pH 9.0 due to the formation of sparingly soluble species. All of this experimental evidences suggest that at neutral pH only the hydroxo-oxo part of the ligand is involved in the formation of the expected species in these conditions, namely Zn(*L5*)H^2+^, whereas at higher pH the whole *L5* structure is involved in the Zn(*L5*)^+^ formation. As usual, the theoretical chemical shifts, due to the nuclei belonging to each single complex species, were calculated by means of HypNMR (Table S5); once known, they were employed to recalculate the weight average chemical shifts, which should be closer to the observed ones if the proposed speciation model is reliable. From Figure 8, the almost total overlap of the experimental and the calculated average chemical shifts can be clearly observed, shown here for *a*, *c*, *d*, and *f* nuclei of *L5* in the Zn^2+^/*L5* system, thus confirming the model employed for the rationalization of the data coming from potentiometric and spectrophotometric investigations. 

**Figure 9:**
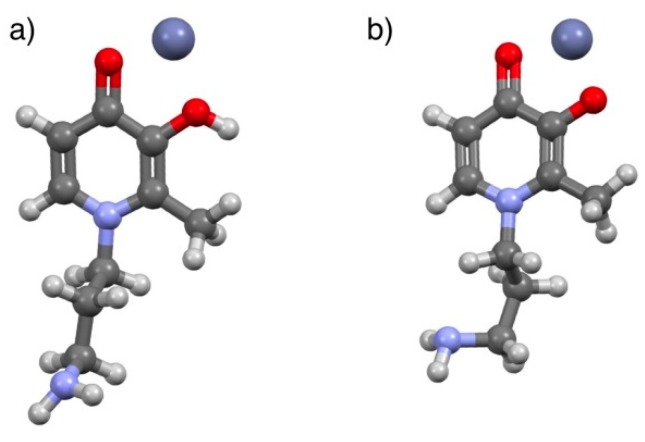
Ball-and-stick views of the DFT-optimized geometry (B3LYP/6-31G(d)) of the Zn^2+^/*L5* species: (**a**) Zn(*L5*)H^2+^ and (**b**) Zn(*L5*)^+^. Oxygen = red; carbon = grey; hydrogen = white, nitrogen = blue; zinc = dark blue.

The geometry of the Zn(*L5*)H^2+^ and Zn(*L5*)^+^ species was obtained by performing a preliminary equilibrium conformer search with a molecular modelling force field (MMFF). The minimum energy conformers were refined by the PM6 (Parameterization Method 6) semiempirical method, and the resulting structures were used as inputs for a geometry optimization at the DFT level of theory (B3LYP/6-31G(d)) [29]. Both complexes returned a similar arrangement (Figure 9), with the Zn^2+^ cation interacting with both the oxygen atoms of the pyridinone ring, again confirming the conclusions drawn by the ^1^H NMR experiments. The only significant difference between the two complexes was in the oxygen–metal distances, which were found to be longer in Zn(*L5*)H^2+^ (=O···Zn^2+^···OH of 1.85 and 2.04 Å, respectively) than in Zn(*L5*)^+^ (=O···Zn^2+^···O^–^ of 1.85 and 1.84 Å, respectively), probably owing to the additional electrostatic attraction in the latter species.

Potentiometric studies were also performed at different ionic strengths (0.15 ≤ *I*/mol L^−1^ ≤ 1.00) and temperatures (288.15 ≤ *T*/K ≤ 310.15) to check the influence of these variables on the speciation. In Table 3, the formation constants determined at the different experimental conditions are reported.

As also observable in Figure S9I, the log*β*_110_ values decrease with *I* and *T* increasing. The same behavior characterizes the log*K*_110_ trend with temperature increase, while its stability decreases with ionic strength increasing, and it shows an inversion tendency at *I* = 1.00 mol L^−1^. The variables effect can also be increased, as seen by the distribution diagrams in Figure S9II, drawn for two conditions: ionic strength increase, namely *I* = 0.161, 0.472, 0.951 mol L^−1^, *T* = 298.15 K; and temperature increase, *I* = 0.15 mol L^−1^, *T* = 288.15 K, and *T* = 310.15 K. In the first case, the Zn^2+^/3-hydroxypyridinone complexation starts at pH ~ 3.0 and the percentages of Zn(*L5*)H^2+^ species increase with ionic strength, reaching values of 67%, 81%, and 92% in the pH range of 6.6–6.9. The metal–ligand 1:1 stoichiometry species formation, instead, is shifted at more alkaline pH conditions with *I* increase. In the second case, similarly to the previous system, metal complexation is favored at high temperature, beginning at pH < 2.0. The main species at physiological pH levels is Zn(*L5*)H^2+^, where the formation of hydrolytic Zn(OH)_2_^0^_(aq)_ and Zn(OH)_3_^−^ species occur, which does not happen at lower temperatures.

##### Confirmation of Zn^2+^/*L5* Species Formation by ESI-MS

In the literature, Clarke et al. [30] reported the results of a potentiometric and UV-Vis spectrophotometric studies on the speciation of deferiprone, a 1,2-dimethyl-3-hydroxy-4-pyridinone ligand, in the presence of trivalent and divalent metal cations, including Zn^2+^ at *I* = 0.15 mol L^−1^ in KCl_(aq)_ and *T* = 298.15 K. In the case of zinc, two complexes were obtained, namely a 1:1 and a 1:2 stoichiometry metal–ligand species. On the contrary, Grgas-Kužnar’s group [31] reported the results of an investigation on a Zn^2+^/dopamine system at *I* = 0.50 mol L^−1^ in NaNO_3(aq)_ and *T* = 303.15 K, determining only the Zn(Dop)H^+^ species. Dopamine is characterized by a similar structure to *L5*, namely by an aromatic ring with two –OH substituents in the ortho position and an ethylamine group. As already discussed, the study presented in this paper on Zn^2+^/3-hydroxy-pyridinone systems, did not lead to the determination of the 1:2 stoichiometry metal–ligand species, at the experimental conditions of the measurements, performed with different analytical techniques.

In light of these considerations, amongthe Zn^2+^/(3-hydroxy-4-pyridinone) systems, the Zn^2+^/*L5* system was selected for further studies. Therefore, with the aim of confirming the Zn(*L5*)^+^ complex formation and further verifying the possible determination of a Zn(*L5*)_2_^0^_(aq)_ species, ESI-MS (Electrospray mass) measurements were performed on samples at different ionic strength conditions, such as *I* = 0.15 and 1.00 mol L^−1^ in NaCl_(aq)_ at pH ~ 10.5. This pH value was chosen because it represents a condition under which only the 1:1 stoichiometry metal–ligand species is present in solution (see Figure 9), taking into account the speciation model already reported. The focus on this pH is also significant because the formation of the 1:2 stoichiometry metal–ligand species may occur in approximately the same pH range. Some measurements were also carried out in the absence of ionic medium to make comparisons among the possible species formed for NaCl-containing systems.

Prior to investigating the metal–ligand complexation, the spectra of ligand and metal cation were studied separately at the already cited experimental conditions. As observable in the distribution diagrams for *L5* reported in Figure S5, and in accordance with the data already reported by this research group [8], the monoprotonated ligand species is present in solution at pH ~ 10.5, together with the fully deprotonated one. From the analysis of ESI-MS spectra recorded at all selected experimental conditions, the formation of three main ligand species was noticed, with *m/z* = 183.1, 205.1, and 221.1 (Table 4), attributable to the [*H*(*L5*) + H]^+^ species and to the [H(*L5*) + Na]^+^ and [*H*(*L5*) + K]^+^ adducts, respectively. 

The observation of the sodium and potassium adducts is very common in ESI-MS spectra, possible due to background impurities and to the considerable presence, in some cases, of ionic medium NaCl in solution [32]. Furthermore, an intensity decrease of the [*H*(*L5*) + H]^+^ peak with ionic strength increase was noticed, in particular at *I* = 1.00 mol L^−1^, due to a signal suppression attributable to the ionic medium effect, leading to a significant salt-adducted species formation [33,34,35]. 

Regarding the metal behavior, firstly it was investigated at pH ~ 10.5, but under all experimental conditions (*I* = 0.15, 1.00 mol L^−1^ in NaCl_(aq)_, absence of ionic medium) the formation of a white, sparingly soluble species, probably attributable to Zn(OH)_2_^0^_(s)_, made this investigation difficult. For these reasons, we decided to carry out some measurements at similar experimental conditions to the literature ones in the pH range of 6.0–7.5, in order to gain information on the ESI-MS Zn^2+^ behavior in aqueous solution and make comparisons with some data already present in the literature. ZnI_2_ (*c*_Zn2+_ = 0.002 mol L^−1^) was used instead of ZnCl_2_ as a source of zinc and in the absence of ionic medium, however formation of the sparingly soluble species was not observed. At this pH range, as observable in Figure S10, the distribution diagram of Zn^2+^ in the absence of ionic medium, drawn using literature hydrolytic constants [6,25,26], shows that the metal cation is almost totally present in the free form, together with low percentages (<10%) of Zn(OH)^+^ species. In accordance with the literature data [36], from the analysis of ESI-MS Zn^2+^ spectra, peaks related to the formation of [Zn(H_2_O)_n_]^2+^ and [Zn(OH)(H_2_O)_n_]^+^ clusters were noticed, as well as those attributable to chloride and sodium adducts, as reported in Table 4. 

Concerning the Zn^2+^/*L5* complexation, the analysis of ESI-MS spectra, recorded at *I* = 0.15 and 1.00 mol L^−1^ in NaCl_(aq)_, and in the absence of ionic medium, showed the presence of peaks related to *L5* in all cases (i.e., [H(*L5*) + H]^+^, [H(*L5*) + Na]^+^, and [H(*L5*) + K]^+^ adducts). In addition, the formation of the 1:1 stoichiometry metal–ligand complex was detected in the form of an adduct cluster with water molecules, Na^+^, and Cl^−^ ions, caused either by the background or the ionic medium effect (Table 4). Similar to the investigation of *L5* spectra, also in this case the effect of NaCl was significant, and a signal suppression of peaks with ionic strength increasing characterized the complex under study. Moreover, regarding the possible formation of the 1:2 stoichiometry metal–ligand species, a small peak with a value of *m/z* = 448.9 was noticed only in the spectra in the absence of ionic medium, and was not present in any of those recorded at *I* = 0.15 and 1.00 mol L^−1^. This signal, perhaps, could be attributable to a possible adduct [Zn(*L5*)_2_ + Na]^+^ with a theoretical value of *m/z* = 449.11, but the difference between the theoretical and the experimental values seems excessive for the type of instrument used.

Actually, on light of this consideration, and taking into account that all the results reported in the present paper were obtained from measurements in the presence of NaCl, we feel confident to state that under the used experimental conditions, the 1:2 stoichiometry metal–ligand species does not form, but we cannot exclude its formation under other conditions.

### 2.5. Dependence on Ionic Strength and Temperature

The dependence on ionic strength and temperature of *L2* and *L5* protonation and stability constants towards Zn^2+^ in NaCl_(aq)_ was modeled using Equations (7)–(9). The thermodynamic parameters (equilibrium constants atinfinite dilution, C and Δε empirical parameters, Δ*H* values), determined by taking into account the values obtained from UV-Vis spectrophotometric, spectrofluorimetric [8], potentiometric, and ^1^H NMR measurements, are reported in Table 5, together with the overall and stepwise free energy (Δ*G*) and the entropy changes (*T*Δ*S*). The assessment of thermodynamic parameters provides a complete picture of the systems, whose equilibrium constants can be predicted for any experimental conditions. 

The stepwise protonation enthalpy changes calculated at *I* = 0.15 mol L^−1^ and *T* = 298.15 K resulted in good agreement among the protonable groups belonging to both the ligands under investigation, and in all cases the reactions were spontaneous (Δ*G* < 0). The protonation of –OH groups resulted an endothermic process, with positive Δ*H* values and a consequent heat absorbance from the system environment, while the entropic contribution was the driving force for the formation of protonated species. On the contrary, the protonation of the remaining sites is exothermic in nature, with negative Δ*H* values and a resulting heat transfer from the system environment. The comparison with the calculated *T*Δ*S* values allowed us to assert that for –NH_2_, –COOH, and pyridinone nitrogen atoms, the enthalpic factor provided the driving force for the formation of protonated species. In the case of Zn^2+^/3-hydroxy-pyridinone interactions, as listed in Table 5, for both ligands the enthalpic contribution is the main driving force for the complex formation of Zn*L*H^(3−z)^ and Zn*L*^(2−z)^ species, which is exothermic in nature; Zn(*L2*)OH^−^ is instead characterized by an opposite tendency.

### 2.6. Literature Data Comparison

#### 2.6.1. Protonation of the Ligands

The acid–base properties of *L2* and *L5*, reported in this paper in NaCl_(aq)_ at different ionic strength and temperature conditions, can be compared with data published in the literature by our research group on the same two ligands at *I* = 0.15 mol L^−1^ in NaCl_(aq)_ and at *T* = 298.15 K and 310.15 K [8], and also by other authors for products (see Figure S11) characterized by similar structures and functional groups. In particular, it is possible to make the comparison of the two ligands’ protonation constants at *I* = 0.15 mol L^−1^ in NaCl_(aq)_ and *T* = 298.15 K (Table 1), withdata from Clevette and coworkers [5], Crisponi’s group [37,38,39], and Clarke et al. [30] (listed in Table 6). The first author worked at the same experimental conditions reported above, while the second and the third groups worked at conditions of *I* = 0.15 mol L^−1^ in KCl_(aq)_ and *T* = 298.15 K. Bretti et al. [40] published a complete study on deferiprone (DFP) behaviour in NaCl aqueous solution, studied by means of potentiometric measurements carried out at 0.10 ≤ *I*/mol L^−1^ ≤ 4.92 and 283.15 ≤ *T*/K ≤ 318.15.

The DFP protonation constants (Table 6), refined by the author at quite similar *I* and *T* conditions to those used in the present work, are in good agreement with the values obtained here for H(*L2*)^−^, H_4_(*L2*)^2+^, H(*L5*)^0^_(aq)_, and H_3_(*L5*)^2+^ species, as reported in Table 1. As already mentioned in the literature [8] and in the section detailing *L5* ligand acid–base properties, H(*L2*)^−^ and H(*L5*)^0^_(aq)_ protonation constants are related to the –OH groups, while the H_4_(*L2*)^2+^ and H_3_(*L5*)^2+^ ones are related to the pyridinone nitrogen atom protonation. 

The same research group [41] also reported the results of a potentiometric investigation carried out on pyridine in NaCl_(aq)_, 0.29 ≤ *I*/mol L^−1^ ≤ 3.60, and at 288.15 ≤ *T*/K ≤ 310.15. In this case, the constant related to the nitrogen atom protonation showed similar ionic strength trends to those obtained for both the studied *L2* and *L5* ligands, but at similar experimental conditions to those reported here, the values reported by the author were higher than the ones we found. This difference may be due to a different electronic charge delocalization and to the absence on the pyridine ring structure of substituents and alkylic chains, which could influence the acid–base properties of the ligand. This research group [24] also investigated dopamine (Figure S11) behaviour in NaCl aqueous medium by means of potentiometric, UV-Vis spectrophotometric, and spectrofluorimetric measurements performed at 0.15 ≤ *I*/mol L^−1^ ≤ 1.02 and 288.15 ≤ *T/*K ≤ 318.15, where two protonable groups were determined, possibly related to the substituent groups, namely –OH in the aromatic ring and –NH_2_ on the alkyl chain, which were present on the molecules. The affinity between dopamine data (Table 6) and *L2* and *L5* protonation constants (Table 1) at the different experimental conditions could be explained by the similarity of some dopamine moieties with respect to the ligand structures under investigation. Analogous data were obtained by Grgas-Kužnar’s group [31], using a potentiometric study performed at *I* = 0.50 mol L^−1^ in NaNO_3(aq)_ and *T* = 303.15 K. In this case, the only difference was the protonation constant value related to the other –OH group present on the molecule structure, which was detectable only by carrying out measurements at alkaline pH conditions.

*L2* acid–base properties at different ionic strengths and temperatures can be compared with aspartic acid behavior, from which it is derived, reported by Martell et al. [6] at 0.10 ≤ *I*/mol L^−1^ ≤ 1.00 in Na^+^ ionic medium and *T* = 298.15 K and 310.15 K. The *L2* protonation constants reported in this paper in Table 1 for the –NH_2_ and –COOH groups are in good agreement with the literature data (Table 6), also providing a log*K*^H^ value for a second carboxylic group, which is not present in our structure. 

Lastly, as already mentioned in the sections detailing *L2* and *L5* behavior in aqueous solution, there was a good accordance among the –NH_2_ groups’ protonation constants at *I* = 0.15 mol L^−1^ in NaCl_(aq)_ and *T* = 310.15 K [9], along with the corresponding value for mimosine [23] (Table 6, Figure S11), studied at the same temperature and ionic strength but in KNO_3(aq)_.

#### 2.6.2. Zn^2+^/Ligands Systems

Comparisons can be performed among the data for Zn*L*^(2−z)^ species reported in the literature (Table 7) for similar ligand structures and functional groups as the investigated 3-hydroxy-4-pyridinones.

Clarke et al. [30] published a value of log*K*_110_ = 7.19 at *I* = 0.15 mol L^−1^ in KCl_(aq)_ and *T* = 298.15 K, which is similar to the corresponding one here obtained for the Zn^2+^/*L1* system at *I* = 0.15 mol L^−1^ in NaCl_(aq)_ and *T* = 298.15 K. The authors also determined the formation of a Zn(DFP)^0^_2(aq)_ species, while a hydrolytic mixed species was obtained here with *L1*. 

Analogous to the acid–base properties, comparison can also be made between results and Zn^2+^/pyridine (*L* = Py) complexation data present in the literature. In particular, Desai et al. [42] and Kapinos and coworkers [43] reported the results of potentiometric studies carried out at *I* = 0.10 mol L^−1^ in NaClO_4(aq)_ and *I* = 0.50 mol L^−1^ in NaNO_3(aq)_ and *T* = 298.15 K, respectively, with stability constants values of about 7–8 orders of magnitude lower than those we obtained for the Zn*L*^(2−z)^ species, as could be expected due to the absence of functional groups that may participate in the metal coordination. The first author reported a speciation model characterized by protonated, simple 1:1 (Zn(Py)^2+^), 1:2 (Zn(Py)_2_^2+^), and 1:3 (Zn(Py)_3_^2+^) metal–ligand stoichiometry species, while the second one only reported the formation of the Zn(Py)^2+^ complex. 

**Table 7:** Literature stability constants of Zn^2+^/ligands species reported for different temperatures, ionic strengths, and ionic media in molar concentration scale.

Ligand	*I*/mol L^−1^	*T*/K	Ionic Medium	log*K*_111_	log*K*_110_	log*K*_120_	log*K*_130_	Ref.
DFP	0.100	298.15	KCl	-	7.19	6.34	-	[30]
Pyridine	0.100	298.15	NaClO_4_	5.50	1.10	0.60	0.38	[44]
	0.500	298.15	NaNO_3_	-	1.15	-	-	[43]
Dopamine	0.500	293.15	NaNO_3_	7.28	-	-	-	[31]
Aspartic acid	0.100	298.15	NaNO_3_	-	5.35	-	-	[45]
	0.100		Na^+^	-	5.87	-	-	[6]
	0.500		Na^+^	-	5.60	9.93	-	[6]
	1.000		Na^+^	-	5.64	-	-	[6]
	0.100	303.15	Na^+^	-	-	10.16	-	[6]
	0.150	310.15	Na^+^	1.55	5.82	10.13	-	[6]

Grgas-Kužnar’s group [31], as mentioned above, reported the results of a potentiometric investigation carried out on the Zn^2+^/dopamine (*L* = Dop) system at *I* = 0.50 mol L^−1^ in NaNO_3(aq)_ and *T* = 303.15 K. The obtained speciation model was characterized only by the Zn(Dop)H^+^ species, confirming the formation of the monoprotonated complex, as we also obtained for all the metal/–NH_2_-containing ligand group (*L2*–*L5*) systems, which were studied at *I* = 0.15 mol L^−1^ in NaCl_(aq)_ and *T* = 298.15 K. The stability constants found in the literature (Table 7) are ~1–2 orders of magnitude higher than the values presented here, probably because the stability of the formed Zn^2+^/3,4-HPs species can be influenced by the absence of a hydroxyl group and the presence, in some cases, of carboxylic and amidic moieties. 

Sajadi and coworkers [45] published the results of a potentiometric speciation study of Zn^2+^ in the presence of aspartic acid (Asp), of which *L2* and *L3* ligands are derivatives, carried out at *I* = 0.10 mol L^−1^ in NaNO_3(aq)_ and *T* = 298.15 K, while Martell et al. [6] reported data from investigations in Na^+^ ionic medium at different experimental conditions, such as 0.10 ≤ *I*/mol L^−1^ ≤ 1.00 at *T* = 298.15 K, *I* = 0.10 mol L^−1^ at *T* = 303.15 K, and *I* = 0.15 at 310.15 K. In almost all cases, the authors obtained the Zn(Asp)^0^_(aq)_ species, with values 3.5–3.8 orders of magnitude higher than the ones reported here; the formation of Zn(Asp)H^+^ and Zn(Asp)_2_^2−^ species also occurred only at some experimental conditions.

### 2.7. Sequestering Ability

The sequestering ability of a ligand towards a metal cation can be evaluated by the determination of an empirical parameter, pL_0.5_. It has already been proposed by the research group and represents the total concentration of ligand that is required to sequester 50% of a metal cation present in trace concentration in solution. This parameter is described by the following sigmoidal-type Boltzmann equation (Equation (12)):

(12) $$x_{M}=\frac{1}{{1+10}^{\left(\mathrm{pL}-{\mathrm{pL}}_{0.5}\right)}}$$

where *x*_M_ is the mole fraction of the metal cation complexed by the ligand, pL = −log *c*_L_, and pL_0.5_ = −log *c*_L_, if *x*_M_ = 0.5. A detailed description of pL_0.5_ calculation, importance, and applications is reported in the literature [18]. 

An investigation on the sequestering ability of the five 3-hydroxy-4-pyridinone ligands towards Zn^2+^ was carried out at *I* = 0.15 mol L^−1^ in NaCl_(aq)_ and *T* = 298.15 K, and in the case of *L2* and *L5*, also at different ionic strengths (0.15 ≤ *I*/mol L^−1^ ≤ 1.00) and temperatures (288.15 ≤ *T*/K ≤ 310.15). 

**Figure 10:**
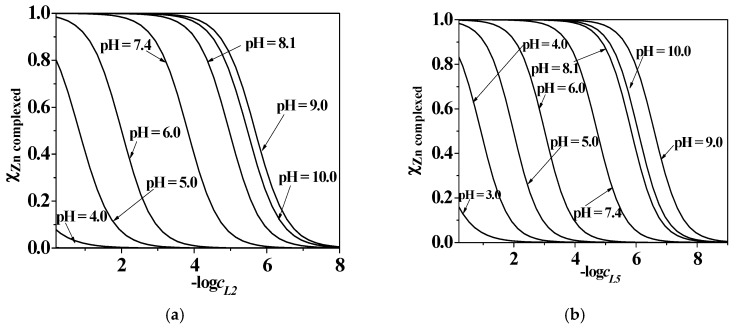

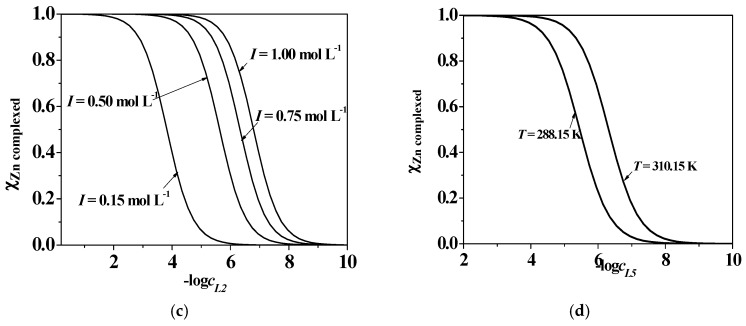
Sequestration diagrams of Zn^2+^/*L2* and *L5* species with different pH (**a**,**b**), ionic strength (**c**), and temperature (**d**) values.

From the data reported in Table 8 and the graphs in Figure 10, it can be argued that for all the systems the sequestering ability assumes significant values starting from pH ~ 3.0–4.0, due to the beginning of metal complexation with pH increasing up to pH ~ 9.0, where the possible formation of Zn^2+^ hydrolytic or mixed-hydrolytic species may influence the systems. At physiological pH level (pH ~ 7.4), *I* = 0.15 mol L^−1^ in NaCl_(aq)_, and *T* = 298.15 K, the sequestering ability is influenced, as expected, by the different acid–base properties of the ligands, and it decreases with alkyl chain length; the trend is: *L1* > *L5* > *L4* > *L3* > *L2.* Concerning the ionic strength effect, as already stated for *L2* protonation behaviour, the presence of sodium chloride would seem to stabilize the system, which may be the reason why, as shown in Table 8 and Figure 10, the sequestering ability increases with increasing *I*, while in the case of Zn^2+^/*L5* species, it reaches its maximum value at *T* = 310.15 K.

### 2.8. Zn^2+^ Depletion vs. Al^3+^ Sequestration

A comparison of this paper’s data can be performed with those already reported in the literature by this research group for the Al^3+^ complexation by the same bifunctional ligands [8], at *I* = 0.15 mol L^−1^ in NaCl_(aq)_ and *T* = 298.15 K. The main purpose of this paper, as already mentioned, was to verify, from a thermodynamic point of view, if there could be a significant depletion of an important divalent bio-metal such as Zn^2+^, along with the strong sequestering activity towards the trivalent metal cations. In particular, the determined stability constants related to the Zn*L*^(2−z)^ species (Table 2) and the corresponding values reported in Table S6 at the same experimental conditions for the Al*L*^(3−z)^ complex can be compared, observing that for all the bifunctional ligands the stability of the simple Al 1:1 stoichiometry species is much higher than those obtained with Zn^2+^. Moreover, a comparison among the physiological pL_0.5_ values calculated for all the ligands towards both the metal cations (Table 8 and Table S6) can be carried out, showing that the sequestering ability of 3-hydroxy-4-pyridinones is stronger towards Al^3+^ than Zn^2+^ for *L1*, *L2*, *L3*, and *L4* ligands, with values of ΔpL_0.5_ = 0.8, 3.9, 2.2, and 3.0, respectively. Only in the case of *L5* is the tendency is opposite, due to the absence of the –COOH group in the ligand structure (Figure 1), which, as already pointed in the literature, should allow the formation of the sparingly soluble Al(OH)_3_^0^_(s)_ species under acidic pH values (pH ~ 5.0), influencing in a noteworthy way the sequestering ability of the ligand towards Al^3+^ [46].

In light of these considerations, it is possible to claim, from a thermodynamic point of view, that no competition between Zn^2+^/Al^3+^ should occur since the ligands promote the removal of *hard* metal cations from the human body, while avoiding the possibility of bio-metal depletion. 

## 3. Materials and Methods 

### 3.1. Chemicals 

HCl and NaOH standard solutions were prepared for dilution of Riedel–deHäen concentrated ampoules and were standardized against sodium carbonate and potassium hydrogen phthalate, respectively. Sodium hydroxide solutions were preserved from atmospheric CO_2_ using soda lime traps. The NaCl ionic medium aqueous solutions were prepared by weighing the pure salt purchased from Fluka, which was previously dried in an oven at *T* = 383.15 K for at least 2 h. The reagents used for all investigations were of the highest available purity and the solutions were prepared with analytical grade water (R = 18 MΩ cm^−1^), using grade A glassware. The ligands under study, namely *L1–L5* (see Figure 1), were synthesized following procedures already reported in the literature [8]. The Zn^2+^ solutions were prepared by weighing ZnCl_2_ Fluka salt without further purification, and standardized using EDTA (ethylenediaminetetraacetic acid) standard solutions; their purity was always ≥98% [47].

### 3.2. Analytical Equipment and Procedures

#### 3.2.1. Potentiometric Tools and Procedure

A Metrohm Titrando (model 809) and a potentiometer with a combined glass electrode (Ross type 8102, from Thermo-Orion) coupled with an automatic burette were used for the potentiometric experiments. This apparatus was connected to a computer and automatic titrations were performed with MetrohmTiAMO 1.2 software, to control titrant delivery, data acquisition, and e.m.f. (electromotive force) stability. Estimated accuracy was ±0.15 mV and ±0.003 mL for e.m.f. and titrant volume readings, respectively. The titrations were carried out using 25 mL of thermostated solution under magnetic stirring. Purified presaturated nitrogen was bubbled into the solutions to keep out the presence of oxygen and carbon dioxide. 

For each measurement, titrations of hydrochloride with standard sodium hydroxide solutions were performed, at the same ionic medium, ionic strength, and temperature conditions as those used for the investigated systems, to refine the electrode potential (E^0^), the acidic junction potential (Ej = *j_a_*[H^+^]), and the ionic product of water (*K*_w_) values. The pH scale used was the free scale and pH≡ −log[H^+^], where [H^+^] is the free proton concentration. Then, 80–100 data points were collected for each titration and the equilibrium state during the experiments was checked by adopting necessary precautions, such as checking the necessary time to reach equilibrium and performing back titrations. 

The acid–base properties and binding ability of *L5* (Figure 1, 5.0·10^−4^ ≤ *c_L5_/*mol L^−1^ ≤ 1.5·10^−3^) towards Zn^2+^ (3.9·10^−4^ ≤ *c*_Zn2+_/mol L^−1^ ≤ 1.0·10^−3^) were investigated by means of potentiometric measurements at metal/ligand ratios between 1:4 and 1:1, 0.15 ≤ *I*/mol L^−1^ ≤ 1.00 in NaCl_(aq)_, 288.15 ≤ *T*/K ≤ 310.15, and in the pH range of 2.0–10.5. Zn^2+^/(*L1*–*L4*) complexation was studied by performing potentiometric measurements at *I* = 0.15 mol L^−1^ in NaCl_(aq)_, *T* = 298.15 K, in the pH range of 2.0–10.5, and with the same concentrations of ligands and metal cations used for the Zn^2+^/*L5* system.

#### 3.2.2. UV-Vis Spectrophotometric Equipment and Procedure

Spectrophotometric measurements were performed by means of an UV-Vis spectrophotometer (Varian Cary 50 model) equipped with an optic fiber probe, featuring a 1 cm fixed-path length. The instrument was linked to a computer and Varian Cary WinUV software was used to record the signal of absorbance (A) vs. wavelength (λ/nm). Simultaneously, potentiometric data were collected using a combined glass electrode (Thermo-Orion Ross type 8102) connected to a potentiometer. A Metrohm 665 automatic burette was used to deliver the sodium hydroxide titrant in 25 mL thermostated cells. A magnetic stirrer ensured the homogeneity of the solutions during the measurements. N_2(g)_ was bubbled in the solutions for at least 5 min before starting the titrations, with the purpose of excluding the possible presence of atmospheric oxygen and carbon dioxide.

UV-Vis spectrophotometric titrations were carried out in NaCl_(aq)_ at 0.15 ≤ *I*/mol L^−1^ ≤ 1.00*,* 288.15 ≤ *T*/K ≤ 310.15, and in the wavelength range of 200 ≤ λ/nm ≤ 800, to study *L2* (4.0·10^−5^ ≤ *c*_L_/mol L^−1^ ≤ 6.0·10^−5^) protonation and binding ability towards Zn^2+^ (2.0·10^−5^ ≤ *c*_Zn2+_/mol L^−1^ ≤ 6.0·10^−5^), with metal/ligand ratios between 1:4 and 1:1. The metal complexation by the other ligands (*L1*, *L3*–*L5*, Figure 1) was investigated by performing measurements at *I* = 0.15 mol L^−1^ in NaCl_(aq),_ *T* = 298.15 K, 2.0 ≤ pH ≤ 10.5. An analogous wavelength range and the concentrations of 3-hydroxy-4-pyridinones and Zn^2+^ used for the Zn^2+^/*L2* system were also selected for these metal–ligands investigations.

#### 3.2.3. ^1^H NMR Apparatus and Procedure

^1^H NMR measurements were recorded using a Bruker AVANCE 300 operating at 300 MHz in 9:1 H_2_O/D_2_O solution. The chemical shifts were measured with respect to 1,4-dioxane and converted relative to tetramethylsilane (TMS), employing δ_(dioxane)_ = 3.70 ppm. The acid–base properties of *L2* have already been investigated and are reported elsewhere [8], whilst *L5* behavior in aqueous solution was studied using ^1^H NMR titrations in 1.0·10^−2^ mol L^−1^ ligand solutions, in the pH range of 2.0–11.0, with *I* = 0.15 mol L^−1^ in NaCl_(aq)_ and *T* = 298.15 K. The spectra of the Zn^2+^-containing solutions in the presence of *L2* or *L5* were recorded by adding known volumes of a sodium hydroxide solution to mixtures of the ligands (5.0·10^−3^ ≤ *c*_L_/mol L^−1^ ≤ 1.0·10^−2^) and the metal cation (2.5·10^−3^ ≤ *c*_Zn2+_/mol L^−1^ ≤ 1.0·10^−2^), in the same ionic medium and pH range already mentioned.

#### 3.2.4. MS Spectroscopy Apparatus and Procedure

Electrospray mass (ESI-MS) spectrometric spectra were recorded on an Agilent LC/MS instrument in positive ion mode using a LC/QQQ 6420 series spectrometer equipped with an electrospray ionization source model G1948B. The instrument was used as simple ESI-MS equipment, thus the column was bypassed and 5 µL of the samples were introduced by flow injection analysis, with water as the solvent flow phase at a flow rate of 0.4 mL min^−1^. Preliminary experiments were performed to establish optimal experimental settings for the ESI-MS conditions, which were capillary voltage 4 kV, fragmentor voltage 135 V, and source temperature 300 °C. Nitrogen was used as the nebulization and desolvation gas at 15 psi and 11 L min^−1^. Spectra were obtained in MS2 scan mode with the Agilent software Masshunter B.06.00.

The mass spectra of *L5*, Zn^2+^, and their mixtures were investigated at *I* = 0.15 and 1.00 mol L^−1^ in NaCl_(aq)_ and in the absence of ionic medium. Ligand and metal concentrations were 1.0·10^−3^ ≤ *c*_L_/mol L^−1^ ≤ 5.4·10^−3^ and 1.0·10^−3^ ≤ *c*_Zn_^2+^/mol L^−1^ ≤ 5.0·10^−3^, respectively. The pH of each measurement solution was adjusted using NaOH standard solutions.

### 3.3. Computer Programs

The determination of the acid–base titration parameters, such as *E*^0^, p*K_w_*, and *j_a_*, and analytical concentration of reagents was carried out using the non-linear least squares ESAB2M [48] computer program. The potentiometric data were elaborated using the BSTAC [49] computer program and checks were performed by means of the HYPERQUAD 2008 software [50]. This last program was also used to analyze UV-Vis spectrophotometric data. Since for *L5* protonation investigations, as well as for Zn^2+^/*L5* and Zn^2+^/*L2* ^1^H-NMR titrations, protons were found to rapidly exchange on the NMR time scale, HypNMR was employed to calculate the individual chemical shifts of all the species present at equilibria, together with the protonation constants, as well as the stability constants [51]. The LIANA [52] computer program was used to refine the parameters for the dependence of thermodynamic parameters on ionic strength and temperature. HySS program [53] allowed the calculation of species formation percentages and the representation of distribution or speciation diagrams. 

### 3.4. Computational Studies

The conformational analysis of the Zn^2+^/*L2* and Zn^2+^/*L5* systems was carried out with the molecular mechanics force field (MMFF) by using the Monte Carlo method to randomly sample the conformational space. 

For the Zn^2+^/*L5* complexes, the equilibrium geometries were further refined using semi-empirical methods (PM6), and finally optimized at the density functional level of theory (DFT, B3LYP functional) using the 6-31G(d) basis set. 

As for the Zn^2+^/*L2* complexes, the conformational analysis was carried out in a water cluster consisting of 100 explicit solvent (water) molecules with the molecular mechanics force field (MMFF), without constraints. The geometry obtained was further refined by semiempirical methods at the PM6 level. All quantum mechanical calculations were performed using Spartan’10 (Wavefunction, Inc., California) [54].

## 4. Conclusions

The acid–base behavior of two bifunctional 3-hydroxy-4-pyridinone ligands, namely *L2* and *L5*, which are derivatives of deferiprone, was investigated by potentiometric, UV-Vis spectrophotometric, and ^1^HNMR titrations at different temperatures and ionic strengths in NaCl_(aq)_. Regarding the protonation of *L2*, the results of UV-Vis studies conducted at different *I* values showed a speciation model with a general trend of protonations occurring at slightly higher pH values with ionic strength, probably due to a noteworthy contribution of the ionic medium to the ligand speciation in aqueous solution. The speciation model obtained for *L5* was confirmed by means of ^1^H NMR spectroscopic titrations performed at *I* = 0.15 mol L^−1^ and *T* = 298.15 K. This analytical technique resulted an effective tool to possibly attribute the protonation constants to the corresponding functional groups. Furthermore, a speciation study of both 3-hydroxy-4-pyridinones in the presence of Zn^2+^ was performed under the same experimental conditions as the protonation study; an investigation at *I* = 0.15 mol L^−1^ and *T* = 298.15 K was also carried out for the other three Zn^2+^/*L*^z−^ systems with ligands belonging to the same class of compounds. The elaboration of the experimental data allowed the determination of overall and stepwise stability constants and metal–ligand species with different stoichiometry (Zn_p_*L*_q_H_r_^(2p+r−qz)^). The obtained values were in accordance with the different analytical techniques and with the data already reported in the literature for ligands with analogous structures and protonable groups. ESI-MS spectrometric measurements performed under different experimental conditions confirmed the formation of 1:1 zinc–*L5* species. Computational studies provided useful information on the geometry of metal–ligand coordination, showing that *L2* binds with Zn^2+^ mainly by its carboxylate moiety, whereas *L5*, in all the pH ranges where zinc–ligand species are formed, takes advantage of the hydroxo-oxo moiety of the pyridinone ring.

The dependence on ionic strength of equilibrium constants was investigated by means of two commonly used models, the extended Debye–Hückel (EDH) model and the classical specific ion interaction theory (SIT). The study of temperature effect, using the van’t Hoff equation, allowed the determination of protonation and formation enthalpy changes calculated at *I* = 0.15 mol L^−1^ and *T* = 298.15 K; the Gibbs free energy and the entropy change were also calculated. For both ligands, the protonation of –OH groups resulted an endothermic process, while an inverse behavior characterized the remaining sites, with reactions being exothermic in nature. Regarding Zn^2+^/3-hydroxy-pyridinone interactions, the enthalpic contribution resulted in the main driving force for the complex formation of Zn*L*H^(3−z)^ and Zn*L*^(2−z)^ species, which was exothermic in nature; the formation of Zn(*L2*)OH^−^ was instead characterized by an opposite tendency. 

Furthermore, on the light of the comparison between the Zn^2+^ data reported in the present paper and those publishedin the literature for Al^3+^/3,4-HP complexation, from a thermodynamic point of view it is possible to affirm that no competition between Zn^2+^/Al^3+^ should occur, since the ligands are able to promote the removal of the *hard* metal cation from the human body, while avoiding the possibility of bio−metal (zinc) depletion. At physiological pH, *I* = 0.15 mol L^−1^ in NaCl_(aq)_, and *T* = 298.15 K, the sequestering ability follows the trend: *L1* > *L5* > *L4* > *L3* > *L2*; in addition, it increases with *I* increasing, while for Zn^2+^/*L5* species, it reaches its maximum value at *T* = 310.15 K.

## Figures and Tables

**Table 1:** Experimental overall ^1^ and stepwise ^2^ protonation constants of *L2* and *L5* ligands at different temperatures and ionic strengths in NaCl_(aq)._

Ligand	*I*/mol L^−1^	*T*/K	log*β*_r_^H 1^ (log*K*_r_^H^) ^2^			
			H*L*^(1−z)^	H_2_*L*^(2−z)^	H_3_*L*^(3−z)^	H_4_*L*^(4−z)^
*L2* ^3^	0.149	288.15	10.28 ± 0.01^4^	19.56 ± 0.01 ^4^ (9.28)	24.29 ± 0.08 ^4^ (4.73)	27.46 ± 0.08 ^4^ (3.17)
	0.506	298.15	9.962 ± 0.005	17.63 ± 0.03 (7.668)	22.52 ± 0.02 (4.89)	25.62 ± 0.02 (3.10)
	0.744	298.15	9.882 ± 0.008	17.25 ± 0.07 (7.365)	22.18 ± 0.10 (4.93)	25.41 ± 0.10 (3.23)
	1.012	298.15	10.059 ± 0.001	16.78 ± 0.02 (6.721)	21.18 ± 0.03 (4.40)	24.91 ± 0.12 (3.73)
*L5* ^5^	0.166	288.15	10.53 ± 0.03 ^4^	19.92 ± 0.02 ^4^ (9.39)	23.58 ± 0.03 ^4^ (3.66)	-
	0.165	298.15	10.82 ± 0.07	20.44 ± 0.06 (9.62)	24.02 ± 0.07 (3.58)	-
	0.140	298.15	11.20 ^6^ ± 0.02	20.466 ^6^ ± 0.006 (9.266)	23.679 ^6^ ± 0.007 (3.213)	-
	0.473	298.15	9.99 ± 0.01	19.17 ± 0.01 (9.18)	22.46 ± 0.01 (3.29)	-
	0.723	298.15	9.79 ± 0.03	18.86 ± 0.03 (9.07)	22.02 ± 0.04 (3.16)	-
	1.008	298.15	10.31 ± 0.02	19.32 ± 0.03 (9.01)	23.38 ± 0.06 (4.06)	-

Note: ^1^ log*β*_r_^H^ refers to Equation (2); ^2^ log*K*_r_^H^ refers to Equation (1); ^3^ data obtained by means of ultraviolet-visible (UV-Vis) spectrophotometric measurements; ^4^ standard deviation; ^5,6^ protonation constants determined by potentiometric and ^1^H NMR titrations, respectively.

**Table 2:** Overall ^1^, stepwise ^2^ experimental, and average ^3^ stability constants of Zn^2+^/3-hydroxy-4-pyridinone species obtained by potentiometric ^4^, UV-Vis spectrophotometric ^5^, and ^1^H NMR ^6^ spectroscopic measurements at *I* = 0.15 mol L^−1^ in NaCl_(aq)_ and *T* = 298.15 K.

	**log*β*_pqr_^1^** **(log*K*_pqr_) ^2^**	**log*β*_pqr_^1^** **(log*K*_pqr_) ^2^**					**log*β*_pqr_^1^** **(log*K*_pqr_) ^2^**
*I*/mol L^−1^	0.146	0.147	0.150	0.145	0.150		0.148
**Species**	***L1***	***L2***					***L3***
Zn*L*H^(3−z)^	-	15.50 ^4^ ± 0.06 ^7^ (4.77)	15.51 ^5^ ± 0.08 ^7^(4.78)	15.79 ^6^ ± 0.31 ^7^ (5.05)	15.56 ^3^ ± 0.08 ^8^ (4.83)		17.06 ^4^ ± 0.09 ^6^ (6.10)
Zn*L*^(2−z)^	7.27 ^4^ ± 0.03 ^7^	8.12 ± 0.06	8.11 ± 0.04	8.54 ± 0.48	8.20 ± 0.10		9.52 ± 0.06
Zn*L*(OH)^(1−z)^	0.25 ± 0.07 (9.45) ^9^	−0.72 ± 0.09 (8.48) ^9^	−0.55 ± 0.04 (8.64) ^9^	−0.72 ± 0.09 (8.64) ^9^	−0.68 ± 0.06 (8.56) ^9^		0.70 ± 0.08 (9.90) ^9^
	**log*β*_pqr_^1^** **(log*K*_pqr_) ^2^**			**log*β*_pqr_^1^** **(log*K*_pqr_) ^2^**			
I/mol L^−1^	0.145	0.150	0.147	0.149	0.150	0.145	0.150
**Species**	***L4***			***L5***			
Zn*L*H^(3−z)^	16.69 ^4^ ± 0.05 ^7^ (5.59)	16.68 ^5^ ± 0.20 ^7^ (5.58)	16.68 ^3^ ± 0.02 ^8^ (5.58)	17.21 ^4^ ± 0.06 ^7^ (6.13)	17.41 ^5^ ± 0.07 ^7^ (6.33)	17.64 ^6^ ± 0.08 ^7^ (6.56)	17.46 ^3^ ± 0.09 ^8^ (6.38)
Zn*L*^(2−z)^	9.68 ± 0.04	9.68 ± 0.10	9.68 ± 0.08	9.89 ± 0.08	9.08 ± 0.02	9.04 ± 0.04	9.22 ± 0.20
Zn*L*(OH)^(1−z)^	0.60 ± 0.09 (9.80) ^9^	0.33 ± 0.20 (9.53) ^9^	0.46 ± 0.08 (9.66) ^9^	-	-	-	-

Note: ^1^ log*β*_pqr_ refers to Equation (5); ^2^ log*K*_pqr_ refers to Equation (4); ^3^ values obtained by averaging potentiometric, UV–Vis spectrophotometric, and ^1^H NMR spectroscopic data; ^4,5,6^ log*β*_pqr_ determined by potentiometry, UV-Vis spectrophotometry, and ^1^H NMR spectroscopy, respectively; ^7^ standard deviation; ^8^ errors on weighed data; ^9^ log*K*_11–1_ refers to equilibrium: Zn(OH)^+^ + *L*^z−^ = Zn*L*(OH)^(1−z)^.

**Table 3:** Overall ^1^ and stepwise ^2^ experimental stability constants of Zn^2+^/*L2* and *L5* species determined at different ionic strengths and temperatures in NaCl_(aq)_.

System	*I*/mol L^−1^	*T*/K	log*β*_111_^1^ (log*K*_111_) ^2^	log*β*_110_^1,2^	log*β*_11−1_^1^ (log*K*_11−1_) ^3^
Zn^2+^/*L2*	0.147	288.15	15.90 ^1^ ± 0.03 ^4^ (5.62)	8.79^1^ ± 0.10^4^	−1.50 ^1^ ± 0.12 ^4^ (8.18)^3^
	0.501	298.15	14.95 ± 0.05 (4.99)	8.34 ± 0.03	−1.26 ± 0.05 (7.82)
	0.759	298.15	14.97 ± 0.10 (5.085)	8.839 ± 0.009	−1.055 ± 0.009 (8.067)
	1.005	298.15	15.47 ± 0.08 (5.44)	9.26 ± 0.01	−0.804 ± 0.003 (8.357)
	0.152	310.15	15.50 ± 0.04 (4.51)	7.93 ± 0.16	0.47 ± 0.04 (9.25)
Zn^2+^/*L5*	0.161	288.15	17.51 ^1^ ± 0.10 ^4^ (6.97)	9.67^1^ ± 0.10^4^	-
	0.472	298.15	16.45 ± 0.02 (6.46)	9.03 ± 0.04	-
	0.725	298.15	16.09 ± 0.04 (6.29)	8.91 ± 0.06	-
	0.951	298.15	16.75 ± 0.09 (6.44)	8.49 ± 0.11	-
	0.155	310.15	16.65 ± 0.10 (6.08)	8.08 ± 0.11	-

Note: ^1^ log*β*_pqr_ refers to Equation (4); ^2^ log*K*_pqr_ refers to Equation (5); ^3^ log*K*_11–1_ refers to equilibrium: Zn(OH)^+^ + *L*^2−^ = Zn*L*(OH)^−^; ^4^ standard deviation.

**Table 4:** ESI-MS signals obtained for *L5*, Zn^2+^, and Zn^2+^/*L5* systems.

Species	Theoretical *m/z*	Experimental *m/z*	Formula
[*H*(*L5*) + H]^+^	183.11	183.1	C_9_H_15_N_2_O_2_
[*H*(*L5*) + Na]^+^	205.10	205.1	C_9_H_14_N_2_NaO_2_
[*H*(*L5*) + K]^+^	221.07	221.1	C_9_H_14_N_2_KO_2_
[Zn(H_2_O)_3_]^2+^	58.98	59.0	ZnH_6_O_3_
[Zn + NaCl]^2+^	60.94	61.0	ZnClNa
[Zn(H_2_O)_5_]^2+^	76.99	76.9	ZnH_10_O_5_
[Zn(OH)]^+^	80.93	81.0	ZnHO
[Zn(OH)(H_2_O)]^+^	98.94	98.9	ZnH_3_O_2_
[Zn(H_2_O)_6_(*L*5) + H + NaCl]^2+^	206.03	206.0	ZnC_9_H_26_N_2_ClNaO_8_

**Table 5:** Thermodynamic parameters for the dependence on ionic strength and temperature of *L2* and *L5* protonation, and Zn^2+^ complex formation in NaCl_(aq)_ at *T* = 298.15 K.

Species	log^T^*β*_pqr_^1^ (log^T^*K*_pqr_) ^2^	C ^3^	Δε ^4^	Δ*H* ^5,6^ (Stepwise Δ*H*)	Δ*G* ^5,6^ (Stepwise Δ*G*)	*T*Δ*S*^5,6^ (Stepwise *T*Δ*S*)
H(*L2*)^−^	11.32 ± 0.08 ^7^	−0.71 ± 0.10 ^7^	−0.69 ± 0.15 ^7^	55 ± 12 ^7^	−61.2 ± 0.2 ^7^	116 ± 4 ^7^
H_2_(*L2*)^0^_(aq)_	20.54 ± 0.02 (9.22)	−2.72 ± 0.07	−2.74 ± 0.15	−200 ± 3 (−255)	−111.4 ± 0.5 (−50.2)	−88 ± 3 (−204)
H_3_(*L2*)^+^	25.35 ± 0.03 (4.81)	−2.94 ± 0.02	−2.88 ± 0.07	−260 ± 4 (−60)	−137.9 ± 0.5 (−26.5)	−122 ± 6 (−34)
H_4_(*L2*)^2+^	28.34 ± 0.04 (2.99)	−2.88 ± 0.02	−2.86 ± 0.10	−269 ± 14 (−9)	−156.5 ± 0.5 (−18.6)	−112 ± 6 (10)
Zn(*L2*)H^+^	16.26 ± 0.12 (4.94)	0.59 ± 0.10	0.64 ± 0.07	−27 ± 9 (−80)	−88.5 ± 0.2 (−27.4)	61 ± 8 (−53)
Zn(*L2*)^0^_(aq)_	8.77 ± 0.05	2.12 ± 0.06	2.01 ± 0.01	−62 ± 1	−46.3 ± 0.2	−16 ± 1
Zn(*L2*)OH^−^	−0.10 ± 0.01 (8.86)	0.25 ± 0.10	0.37 ± 0.05	153 ± 3 (96)	3.0 ± 0.2 (−49.4)	150 ± 3 (146)
H(*L5*)^0^_(aq)_	11.12 ± 0.10 ^7^	−0.83 ± 0.10 ^7^	−0.80 ± 0.19^7^	1 ± 4 ^7^	−63 ± 0.4 ^7^	64 ± 4 ^7^
H_2_(*L5*)^+^	20.54 ± 0.10 (9.42)	−1.26 ± 0.20	−1.24 ± 0.20	−271 ± 2 (−272)	−112.6 ± 1.1 (−49.6)	−158 ± 7 (−222)
H_3_(*L5*)^2+^	23.86 ± 0.08 (3.32)	−1.42 ± 0.20	−1.50 ± 0.32	−322 ± 12 (−51)	−135.1 ± 1.1 (−22.5)	−187 ± 12 (−29)
Zn(*L5*)H^2+^	17.33 ± 0.18 (6.00)	−0.60 ± 0.09	−0.49 ± 0.14	−65 ± 8 (−66)	−97.7 ± 0.3 (−34.7)	33 ± 8 (−31)
Zn(*L5*)^+^	10.57 ± 0.10	−1.24 ± 0.11	−1.38 ± 0.10	−127 ± 15	−52.1 ± 0.8	−75 ± 15

Note: ^1,2^ log^T^*β*_pqr_ and log^T^*K*_pqr_ refer to Equations (1) and (2), and (4)–(6), respectively, and are calculated under infinite dilution; in the molal concentration scale, their values are very similar to the results reported in molar one, with maximum differences of 0.002 logarithmic units; ^3^ parameter determined using Equation (7); ^4^ parameter calculated using Equation (8); ^5^ in kJ mol^−1^; ^6^ at *I* = 0.15 mol kg^−1; 7^ standard deviation.

**Table 6:** Literature protonation constants reported at different temperatures, ionic strengths, and ionic media in molar concentration scale.

Ligand	*I*/molL^−1^	*T*/K	Ionic Medium	log*K*_1_^H^	log*K*_2_^H^	log*K*_3_^H^	log*K*_4_^H^	Ref.
DFP	0.099	283.15	NaCl	10.00	3.80	-	-	[40]
	0.152	298.15	NaCl	9.82	3.67	-	-	[40]
	0.150		NaCl	9.86	3.70	-	-	[5]
	0.495		NaCl	9.70	3.74	-	-	[40]
	1.005		NaCl	9.66	3.83	-	-	[40]
	0.100	310.15	NaCl	9.70	3.60	-	-	[40]
	0.100	298.15	KCl	9.82	3.66	-	-	[39]
	0.150		KCl	9.82	3.66	-	-	[37,38]
	0.150		KCl	9.77	3.68	-	-	[30]
	0.500		KCl	9.78	3.75	-	-	[39]
	1.000		KCl	9.75	3.80	-	-	[39]
	0.100	310.15	KCl	9.70	3.57	-	-	[39]
Pyridine	0.294	283.15	NaCl	5.50	-	-	-	[41]
	0.197	298.15	NaCl	5.28	-	-	-	[41]
	0.480		NaCl	5.37	-	-	-	[41]
	0.960		NaCl	5.52	-	-	-	[41]
	2.018	310.15	NaCl	5.66	-	-	-	[41]
Dopamine	0.147	288.15	NaCl	10.61	9.41	-	-	[24]
	0.162	298.15	NaCl	10.59	9.18	-	-	[24]
	0.504		NaCl	10.44	9.07	-	-	[24]
	0.737		NaCl	10.278	8.99	-	-	[24]
	0.982		NaCl	10.25	8.85	-	-	[24]
	0.165	310.15	NaCl	10.02	8.69	-	-	[24]
	0.500	303.15	NaNO_3_	12.05	10.60	9.06	-	[31]
Aspartic acid	0.100	298.15	Na^+^	9.66	3.71	1.95	-	[6]
	0.500		Na^+^	9.58	3.68	1.98	-	[6]
	1.000		NaCl	9.61	3.64	2.00	-	[6]
	0.150	310.15	Na^+^	9.33	3.64	1.94	-	[6]
Mimosine	0.150	310.15	KNO_3_	8.86	7.00	2.62	1.10	[23]

**Table 8:** The pL_0.5_^1^ values of Zn^2+^/ligand systems with different pH, ionic strengths, and temperatures in NaCl_(aq)_.

System	pH	pL_0.5_	*I*/mol L^−1^	*T*/K	System	pH	pL_0.5_	*I*/mol L^−1^	*T*/K
Zn^2+^/*L1*	2.5	<1.0	0.15	298.15	Zn^2+^/*L2*	7.4	6.3	0.75	298.15
	3.0	<1.0	0.15	298.15		7.4	6.8	1.00	298.15
	4.0	1.0	0.15	298.15		7.4	5.2	0.15	310.15
	5.0	2.5	0.15	298.15	Zn^2+^/*L3*	7.4	4.2	0.15	298.15
	6.0	3.6	0.15	298.15	Zn^2+^/*L4*	7.4	4.4	0.15	298.15
	7.4	5.5	0.15	298.15	Zn^2+^/*L5*	7.4	5.4	0.15	288.15
	8.1	6.7	0.15	298.15		2.5	<1.0	0.15	298.15
	9.0	7.6	0.15	298.15		3.0	<1.0	0.15	298.15
	10.0	7.3	0.15	298.15		4.0	<1.0	0.15	298.15
Zn^2+^/*L2*	7.4	6.3	0.15	288.15		5.0	2.0	0.15	298.15
	2.5	<1.0	0.15	298.15		6.0	3.0	0.15	298.15
	3.0	<1.0	0.15	298.15		7.4	4.7	0.15	298.15
	4.0	<1.0	0.15	298.15		8.1	5.8	0.15	298.15
	5.0	1.0	0.15	298.15		9.0	6.6	0.15	298.15
	6.0	2.0	0.15	298.15		10.0	4.7	0.15	298.15
	7.4	3.8	0.15	298.15		7.4	5.2	0.50	298.15
	8.1	5.0	0.15	298.15		7.4	5.3	0.75	298.15
	9.0	5.7	0.15	298.15		7.4	5.1	1.00	298.15
	10.0	5.5	0.15	298.15		7.4	6.3	0.15	310.15
	7.4	5.6	0.50	298.15					

Values calculated using Equation (12).

## Supplementary Materials

The following are available online, Figures S1–S11, Tables S1–S5.
