# Supplementary material for: Speciation Studies of Bifunctional 3-Hydroxy-4-Pyridinone Ligands in the Presence of Zn2+ at Different Ionic Strengths and Temperatures
Source: Molecules. 2019 Nov 12;24(22):4084. doi: 10.3390/molecules24224084 (PMC6891321; doi:10.3390/molecules24224084)
Supplement: Supplementary file 1 [file molecules-24-04084-s001.pdf]

Supplementary material

Speciation studies of bifunctional 3-hydroxy-4-pyridinone ligands in the presence of Zn^{2+} at different ionic strengths and temperatures

Anna Irto [1], Paola Cardiano [1], Salvatore Cataldo [2], Karam Chand [3], Rosalia Maria Cigala [1], Francesco Crea [1], Concetta De Stefano [1], Giuseppe Gattuso [1], Nicola Muratore [2], Alberto Pettignano [2], Silvio Sammartano [1*] and Maria Amélia Santos [3,*]

[1] Dipartimento di Scienze Chimiche, Biologiche, Farmaceutiche e Ambientali, Università di Messina, Viale F. Stagno d'Alcontres, 31 – 98166 Messina, Italy;
airto@unime.it (A.I.); pcardiano@unime.it (P.C.); rmcigala@unime.it (R.M.C.); fcrea@unime.it (F.C.); cdestefano@unime.it (C.D.S.); ggattuso@unime.it (G.G.)

[2] Dipartimento di Fisica e Chimica Emilio Segrè, ed. 17, Università di Palermo, Viale delle Scienze, I-90128, Palermo, Italy;
salvatore.cataldo@unipa.it (S.C.); nicola.muratore@unipa.it (N.M.); alberto.pettignano@unipa.it (A.P.)

[3] Centro de Química Estrutural, Instituto Superior Técnico, Universidade de Lisboa, Av. Rovísco Pais 1, 1049-001 Lisboa, Portugal;
kc4chemistry@gmail.com (K.C.)

* Correspondence: ssammartano@unime.it (S.S.), Tel.: +39 0906765749; masantos@ist.utl.pt (M.A.S.), Tel.: +351 218419000

Table S1. Overall[a)] and stepwise[b)] protonation constants[a)] of *L1*-*L5* ligands reported in the literature [8] at $I = 0.15$ mol L^{-1} in $NaCl_{(aq)}$, $T = 298.15$ K and 310.15 K

Ligand	T/K	$\log\beta_1^H$	$\log\beta_2^H$ ($\log K_2^H$)	$\log\beta_3$ ($\log K_3^H$)	$\log\beta_4^H$($\log K_4^H$)
L1	298.15	9.947	14.36 (4.41)	17.74 (3.38)	-
	310.15	10.029	13.62 (3.59)	16.54 (2.92)	-
L2	298.15	10.73	19.52 (8.79)	24.17 (4.65)	27.43 (3.26)
	310.15	10.99	17.05 (6.06)	21.02 (3.97)	24.08 (3.06)
L3	298.15	10.93	20.70 (9.77)	25.60 (4.90)	29.02 (3.42)
	310.15	10.93	17.71 (6.78)	22.50 (4.79)	25.93 (3.43)
L4	298.15	11.10	20.44 (9.34)	24.60 (4.16)	27.87 (3.27)
	310.15	11.13	17.93 (6.80)	22.00 (4.07)	25.30 (3.30)
L5	298.15	11.08	20.468(9.388)	23.68 (3.21)	-
	310.15	10.57	16.53 (5.96)	19.53 (3.00)	-

[a)] $\log\beta_r^H$ refer to eq. (2); [b)] $\log K_r^H$ refer to eq. (1); [c)] Std. dev.

Table S2. Overall[a)] and stepwise[b)] protonation constants of *L2* and *L5* ligands at different temperatures and ionic strengths in $NaCl_{(aq)}$ expressed in molal scale (mol (kg $H_2O)^{-1}$)

Ligand	I/mol kg^{-1}	T/K	logβ_r^{H} a) (logK_r^{H}) b)			
			$HL^{(1-z)}$	$H_2L^{(2-z)}$	$H_3L^{(3-z)}$	$H_4L^{(4-z)}$
L2	0.149	288.15	10.28	19.56 (9.28)	24.29 (4.73)	27.45 (3.16)
	0.150	298.15	10.73	19.51 (8.78)	24.16 (4.65)	27.42 (3.26)
	0.512	298.15	9.96	17.62 (7.66)	22.50 (4.88)	25.60 (3.10)
	0.756	298.15	9.87	17.26 (7.39)	22.20 (4.94)	25.44 (3.24)
	1.034	298.15	10.05	16.76 (6.71)	21.15 (4.39)	24.87 (3.72)
	0.151	310.15	10.99	17.04 (6.05)	21.00 (3.96)	24.06 (3.06)
L5	0.167	288.15	10.52	19.92 (9.39)	23.57 (3.65)	-
	0.166	298.15	10.82	20.43 (9.61)	24.01 (3.58)	-
	0.141	298.15	11.20	20.46 (9.26)	23.67 (3.21)	-
	0.151	298.15	11.07	20.46 (9.39)	23.67 (3.21)	-
	0.478	298.15	9.98	19.16 (9.18)	22.44 (3.28)	-
	0.735	298.15	9.78	18.85 (9.07)	22.00 (3.15)	-
	1.030	298.15	10.30	19.30 (9.00)	23.35 (4.05)	-
	0.151	310.15	10.56	16.52 (5.96)	19.52 (3.00)	-

[a)] logβ_r^{H} refer to eq. (2); [b)] logK_r^{H} refer to eq. (1).

Table S3. Calculated chemical shifts of *L2* [8] and *L5* species obtained by ^{1}H NMR titrations at $I = 0.15$ mol L^{-1} in $NaCl_{(aq)}$ and $T = 298.15$ K.

Species	$\delta_{CH\ a}$	$\delta_{CH\ b}$	$\delta_{CH_3\ c}$	$\delta_{CH_2\ d}$	$\delta_{CH_2\ e}$	$\delta_{CH_2\ f1}$	$\delta_{CH_2\ f2}$	$\delta_{CH_2\ g}$
$(L2)^{2-}$	6.24±0.01[a)]	7.05±0.03[a)]	2.30±0.01[a)]	4.08±0.01[a)]	3.52±0.02[a)]	2.47±0.04[a)]	2.64±0.01[a)]	3.39±0.07[a)]
$H(L2)^-$	6.42±0.01	7.45±0.03	2.38±0.01	4.15±0.01	3.52±0.02	2.51±0.04	2.58±0.01	3.57±0.07
$H_2(L2)^0_{(aq)}$	6.47±0.01	7.53±0.03	2.41±0.01	4.18±0.01	3.53±0.02	2.68±0.04	2.79±0.01	3.88±0.07
$H_3(L2)^+$	6.50±0.01	7.54±0.03	2.42±0.01	4.19±0.01	3.54±0.02	2.66±0.04	2.78±0.01	3.86±0.07
$H_4(L2)^{2+}$	7.08±0.01	7.91±0.03	2.58±0.01	4.43±0.01	3.62±0.02	2.83±0.04	2.82±0.01	3.97±0.07
$L5^-$	6.260±0.005[a)]	7.156±0.001[a)]	2.2971±0.0006[a)]	3.982±0.001[a)]	1.783±0.007[a)]	2.535±0.002[a)]	-	-
$H(L5)^0_{(aq)}$	6.360±0.005	7.363±0.001	2.3358±0.0006	4.046±0.001	1.862±0.007	2.652±0.002	-	-
$H_2(L5)^+$	6.491±0.005	7.636±0.001	2.3987±0.0006	4.156±0.001	2.106±0.007	3.026±0.002	-	-
$H_3(L5)^{2+}$	7.128±0.005	8.052±0.001	2.5729±0.0006	4.416±0.001	2.200±0.007	3.087±0.002	-	-

[a)] Std. Dev.

Table S4. Hydrolysis constants[a)] of Zn^{2+} at different ionic strengths in $NaCl_{(aq)}$ and temperatures [6, 33, 34]

Species	288.15 K	298.15 K				310.15 K
	0.15 mol L^{-1}	0.15 mol L^{-1}	0.50 mol L^{-1}	0.75 mol L^{-1}	1.00 mol L^{-1}	0.15 mol L^{-1}
$Zn(OH)^{+}$	-9.68	-9.20	-9.08	-9.12	-9.16	-8.78
$Zn(OH)_2{}^{0}{}_{(aq)}$	-17.92	-17.16	-17.07	-17.15	-17.22	-16.52
$Zn(OH)_3{}^{-}$	-29.51	-28.41	-28.47	-28.47	-28.47	-27.54
$Zn(OH)_4{}^{2-}$	-42.21	-40.66	-40.35	-40.36	-40.38	-39.47
$Zn_2(OH)^{+3}$	-9.45	-8.77	-9.28	-9.38	-8.89	-8.54
$Zn_2(OH)_6{}^{2-}$	-59.66	-57.52	-57.28	-57.30	-57.32	-55.90

[a)] $\log\beta_{pr}$ refer to the equilibrium $pZn^{2+} + rH_2O = Zn_p(OH)_r{}^{(2p-r)} + rH^{+}$.

Table S5. Calculated chemical shifts of Zn^{2+}/*L2* and *L5* species obtained by ^{1}H NMR titrations at $I = 0.15$ mol L^{-1} in $NaCl_{(aq)}$ and $T = 298.15$ K.

Species	$\delta_{CH\ a}$	$\delta_{CH\ b}$	$\delta_{CH_3\ c}$	$\delta_{CH_2\ d}$	$\delta_{CH_2\ e}$	$\delta_{CH_2\ f}$ (*f1* for *L2* ligand)	$\delta_{CH_2\ f2}$	$\delta_{CH_2\ g}$
$Zn(L2)H^{+}$	6.68±0.20[a)]	6.77±0.02[a)]	2.45±0.02[a)]	4.17±0.02[a)]	3.62±0.09[a)]	2.91±0.09[a)]	2.07±0.20[a)]	4.07±0.20[a)]
$Zn(L2)^{0}_{(aq)}$	6.63±0.20	7.24±0.02	2.44±0.02	4.25±0.02	3.19±0.09	2.74±0.09	2.49±0.20	3.75±0.20
$Zn(L5)H^{2+}$	6.651±0.007[a)]	7.37±0.01[a)]	2.450±0.008[a)]	4.21±0.01[a)]	2.144±0.007[a)]	3.03±0.01[a)]	-	-
$Zn(L5)^{+}$	6.624±0.007	7.40±0.01	2.293±0.008	4.01±0.01	1.654±0.007	2.53±0.01	-	-

[a)] ±Std. Dev.

Table S6. Literature stability constants of Al^{3+}/bifunctional 3,4-HPs [8] 1:1 stoichiometry species and $pL_{0.5}$ values (pH = 7.0) reported at I = 0.15 mol L^{-1} in $NaCl_{(aq)}$ and T = 298.15 K and in molar concentration scale

Ligand	$\log\beta_{110}$ [a)]	$pL_{0.5}$
L1	12.57	6.3
L2	17.94	7.7
L3	17.50	6.4
L4	18.32	7.4
L5	15.08	3.9

[a)] $\log\beta_{110}$ refer to equilibrium: $Al^{3+} + L^{z-} = AlL^{(3-z)}$

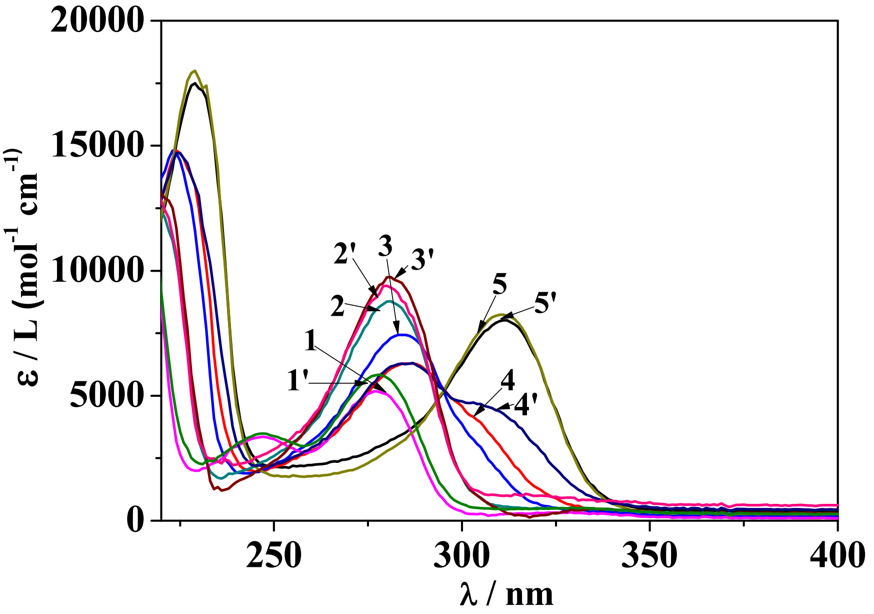

Figure S1. Molar absorbivity of the different *L2* species *vs.* λ at T = 298.15 K in $NaCl_{(aq)}$.

At I = 0.506 mol L^{-1}, species: 1. $H_4(L2)^{2+}$; 2. $H_3(L2)^{+}$; 3. $H_2(L2)^{0}_{(aq)}$; 4. $H(L2)^{-}$; 5. $(L2)^{2-}$.

At I = 1.012 mol L^{-1}, species: 1'. $H_4(L2)^{2+}$; 2'. $H_3(L2)^{+}$; 3'. $H_2(L2)^{0}_{(aq)}$; 4'. $H(L2)^{-}$; 5'. $(L2)^{2-}$.

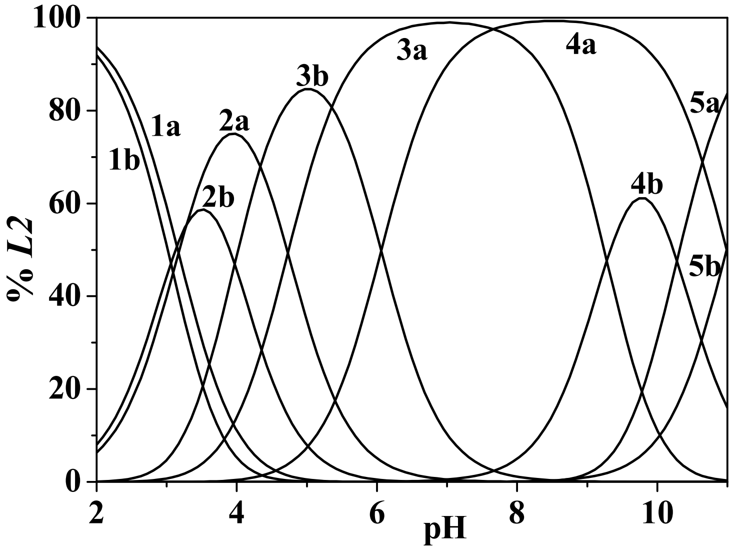

Figure S2. Distribution diagram of *L2* (c_L = 5.3·10^{-5} mol L^{-1}) species at *I* = 0.15 mol L^{-1} in $NaCl_{(aq)}$, *T* = 283.15 K (a) and *T* = 310.15 K (b).

Species: 1. $H_4(L2)^{2+}$; 2. $H_3(L2)^{+}$; 3. $H_2(L2)^{0}_{(aq)}$; 4. $H(L2)^{-}$; 5. $(L2)^{2-}$.

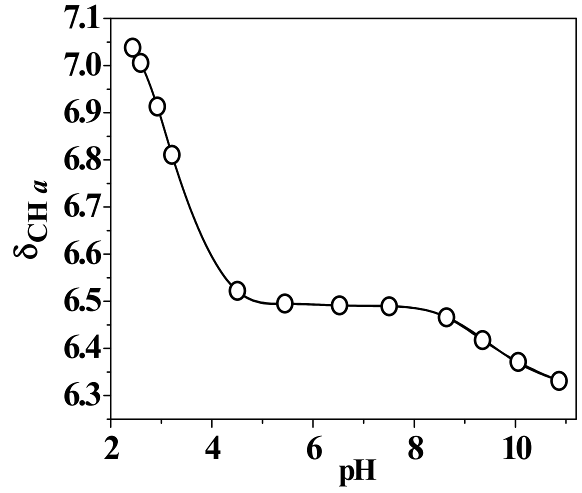

1)

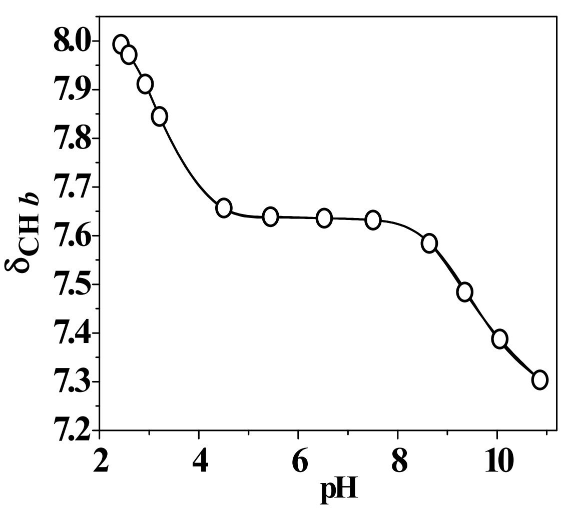

2)

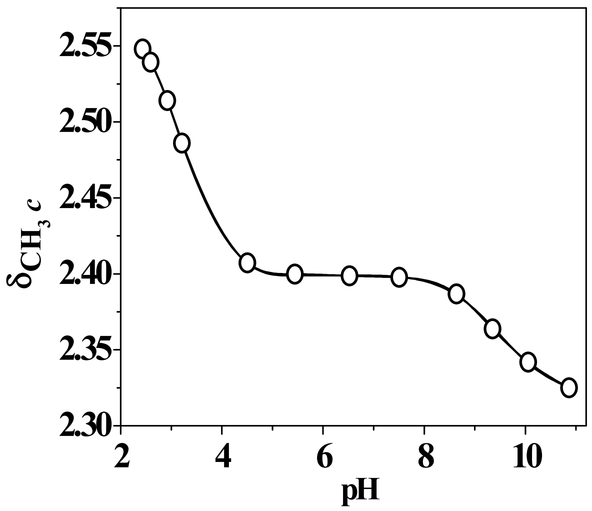

(3)

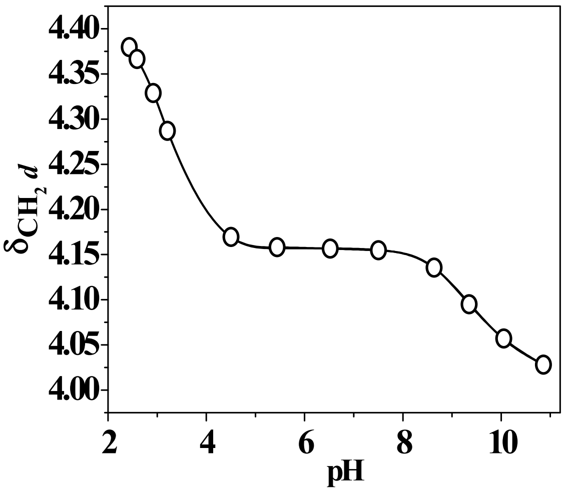

(4)

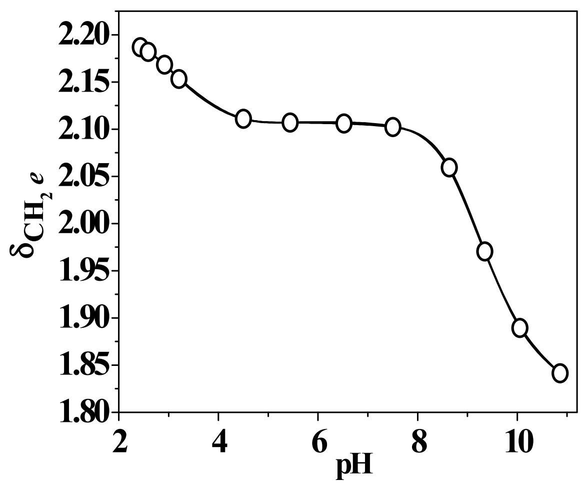

5)

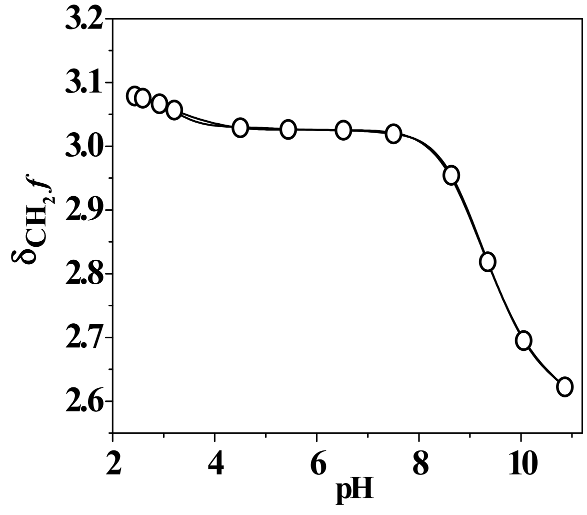

6)

Figure S3. Observed (□) and calculated (○) values of chemical shifts of: a) *a* (1), *b* (2), *c* (3), *d* (4), *e* (5), *f* (6) nuclei of *L5 vs*. pH, at $c_L = 1.0 \cdot 10^{-2}$ mol L^{-1}, $I = 0.15$ mol L^{-1} in $NaCl_{(aq)}$ and $T = 298.15$ K.

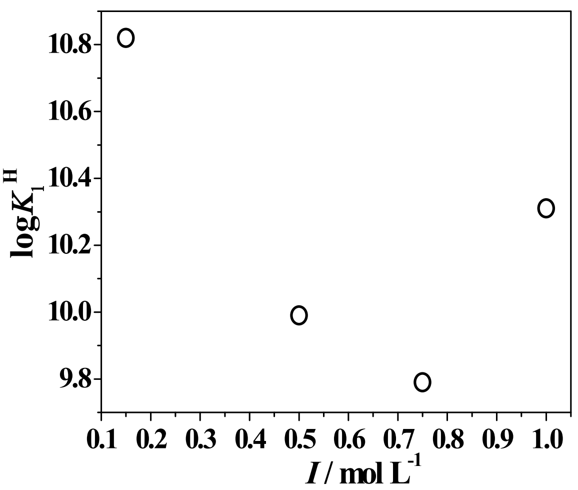

(1)

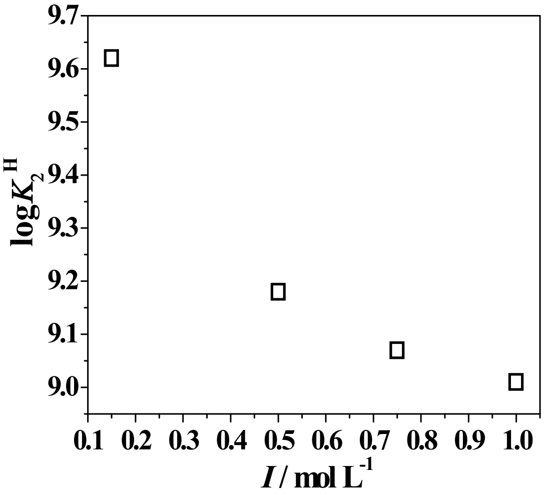

(2)

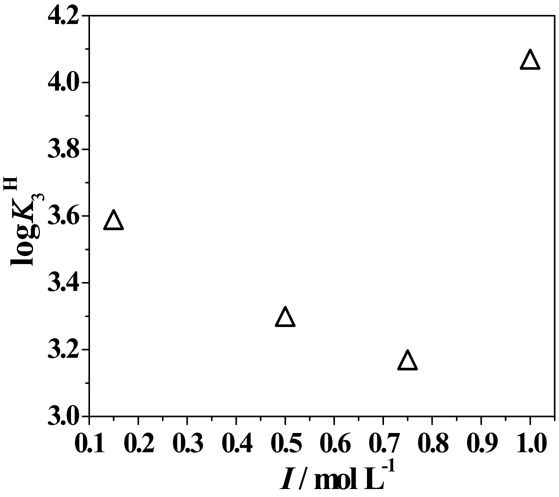

(3)

Figure S4. Trend of logK_1^H (1), logK_2^H (2) and logK_3^H (3) *L5* protonation constants *vs.* the ionic strength (in mol L^{-1}) in $NaCl_{(aq)}$ and T = 298.15 K.

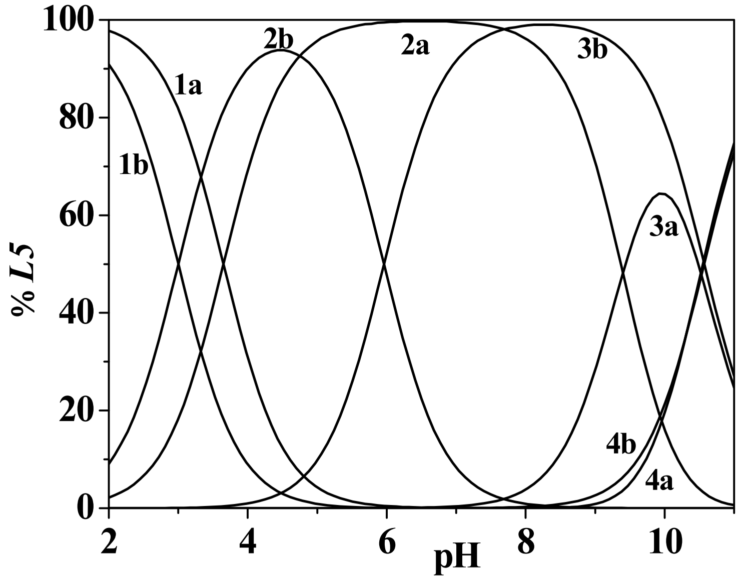

Figure S5. Distribution diagram of *L5* (c_L = 1.0·10^{-3} mol L^{-1}) species at *I* = 0.15 mol L^{-1} in $NaCl_{(aq)}$, *T* = 288.15 K (a) and *T* = 310.15 K (b). Species: 1. $H_3(L5)^{2+}$; 2. $H_2(L5)^{+}$; 3. $H(L5)^{0}_{(aq)}$; 4. $(L5)^{-}$.

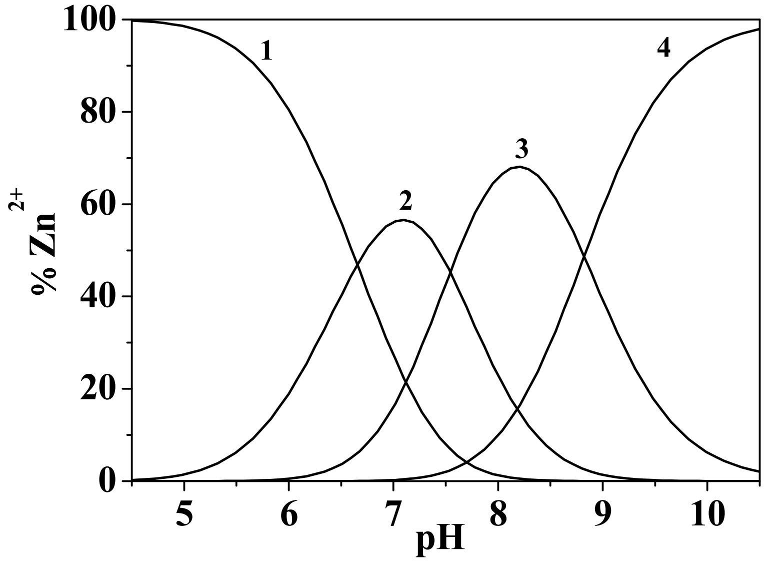

Figure S6. Distribution diagram of Zn^{2+}/*L3* ($c_{Zn^{2+}} = 4.3\cdot10^{-4}$ mol L^{-1}, $c_L = 1.2\cdot10^{-3}$ mol L^{-1}) species at $I = 0.148$ mol L^{-1} in $NaCl_{(aq)}$, $T = 298.15$ K.

Species: 1. free Zn^{2+}; 2. Zn(*L3*)H^+; 3. Zn(*L3*)$^0_{(aq)}$; 4. Zn(*L3*)OH^-.

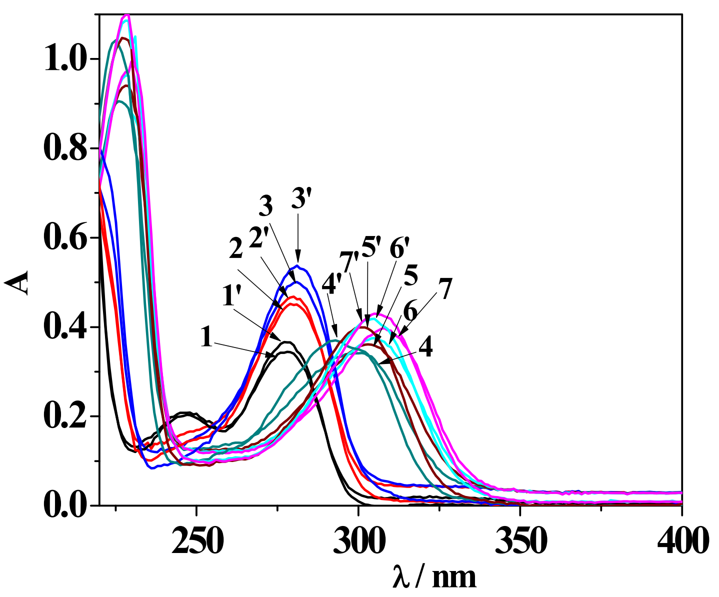

Figure S7. UV-Vis spectrophotometric titration curves of Zn^{2+}/*L2* complexes at $c_{Zn^{2+}} = 2.0\cdot10^{-5}$ mol L^{-1}, $c_L = 5.8\cdot10^{-5}$ mol L^{-1} and different pH values.

At $I = 0.501$ mol L^{-1}: 1. pH = 2.07, λ_{max} = 278 nm; 2. pH = 3.52, λ_{max} = 279 nm; 3. pH = 4.07, λ_{max} = 281 nm; 4. pH = 5.51, λ_{max} = 293 nm; 5. pH = 9.02, λ_{max} = 303 nm; 6. pH = 10.51, λ_{max} = 305 nm; 7. pH = 11.00, λ_{max} = 306 nm.

At $I = 1.005$ mol L^{-1}: 1'. pH = 2.07, λ_{max} = 278 nm; 2'. pH = 3.46, λ_{max} = 279 nm; 3'. pH = 4.15, λ_{max} = 281 nm; 4'. pH = 5.24, λ_{max} = 297 nm; 5'. pH = 8.61, λ_{max} = 300 nm; 6'. pH = 10.65, λ_{max} = 305 nm; 7'. pH = 11.00, λ_{max} = 307 nm.

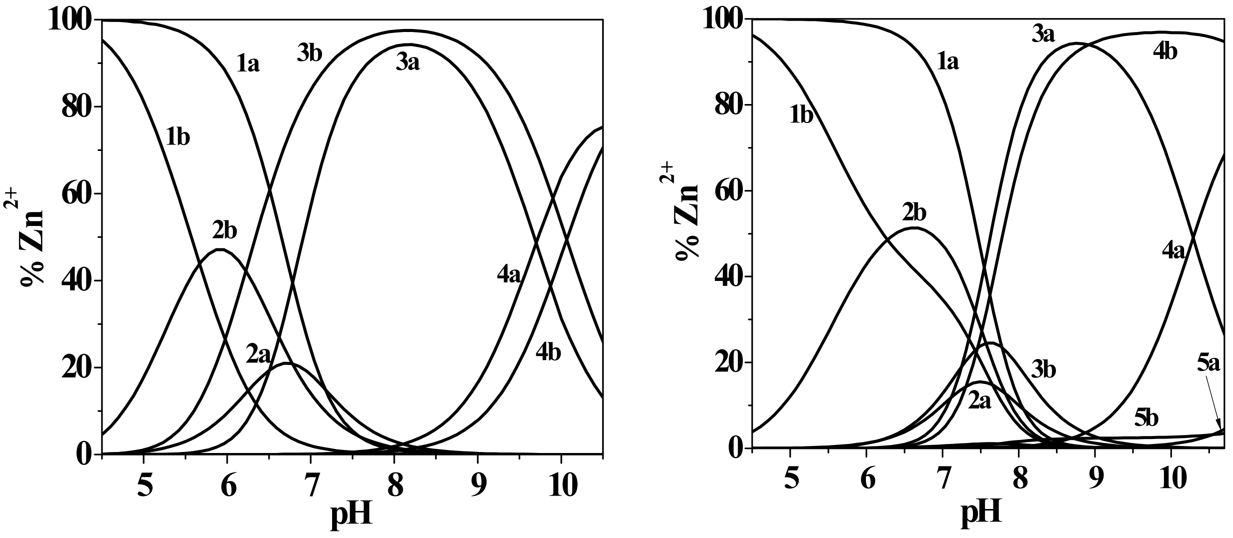

I) II)

Figure S8. Distribution diagram of $Zn^{2+}/L2$ ($c_{Zn^{2+}} = 2.0 \cdot 10^{-5}$ mol L^{-1}, $c_L = 5.8 \cdot 10^{-5}$ mol L^{-1}) species in $NaCl_{(aq)}$ at: **I)** T = 298.15 K and I = 0.501 (a) and 1.005 (b) mol L^{-1}; **II)** I = 0.15 mol L^{-1}, T = 288.15 K and 310.15 K (b). Species: 1. free Zn^{2+}; 2. $Zn(L2)H^+$; 3. $Zn(L2)^0_{(aq)}$; 4. $Zn(L2)OH^-$; 5. $Zn(OH)_2{}^0_{(aq)}$.

I)

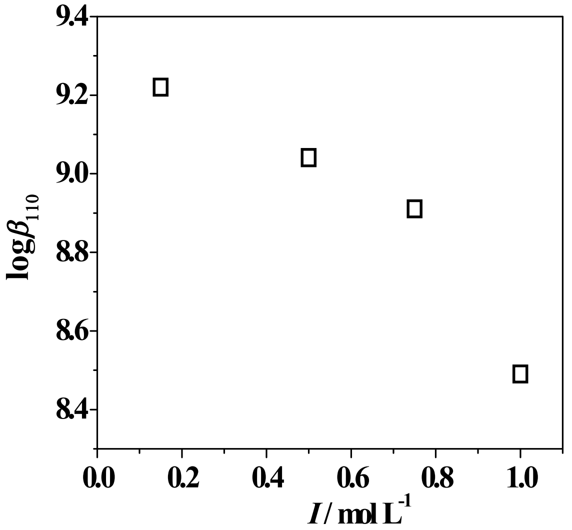

1)

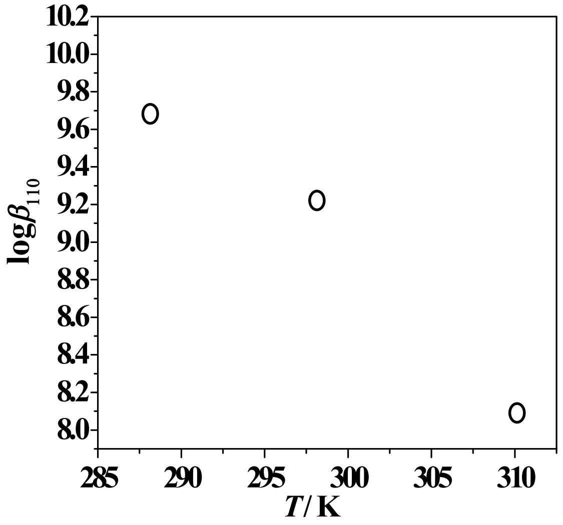

2)

II)

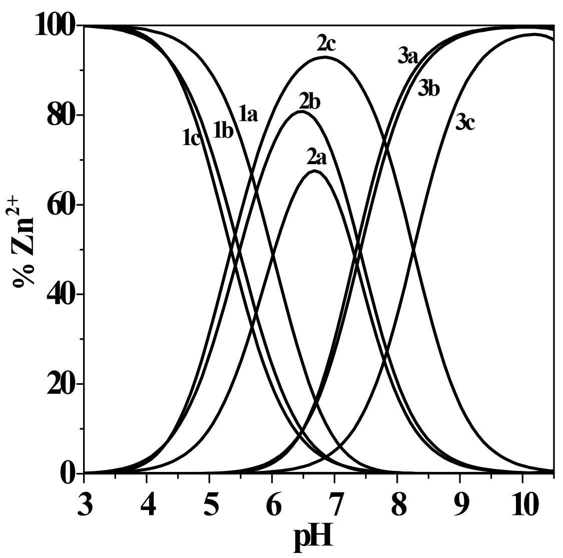

3)

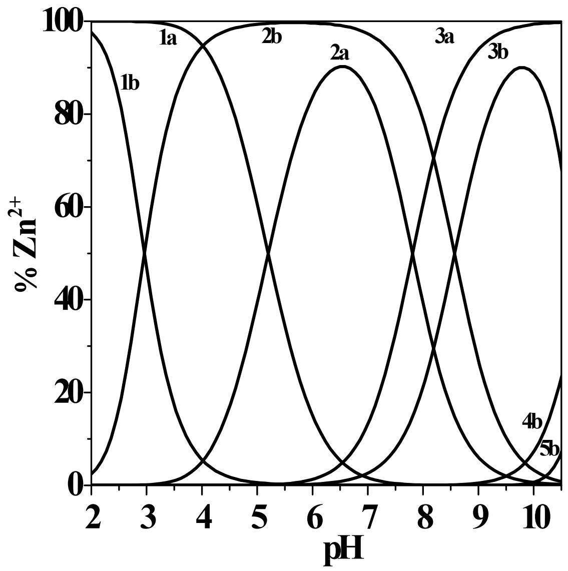

4)

Figure S9. **I)** Trends of Zn^{2+}/*L5* suggested logβ_{110} values *vs.* the ionic strength (in mol L^{-1}) in $NaCl_{(aq)}$ and T = 298.15 K (1) and *vs.* temperature (2) at I = 0.15 mol L^{-1} . **II)** Distribution diagram of Zn^{2+}/*L5* ($c_{Zn^{2+}}$ = 7.0·10^{-4} mol L^{-1}, c_L = 2.1·10^{-3} mol L^{-1}) species in $NaCl_{(aq)}$ at: 3) I = 0.161 (a), 0.472 (b) and 0.951 mol L^{-1} (c) and 298.15 K (b); 4) I = 0.15 mol L^{-1}, T = 288.15 K (a) and T = 310.15 K (b). Species: 1. free Zn^{2+}; 2. Zn(*L5*)H^{2+}; 3. Zn(*L5*)$^{+}$; 4. $Zn(OH)_2{}^0{}_{(aq)}$; 5. $Zn(OH)_3{}^-$.

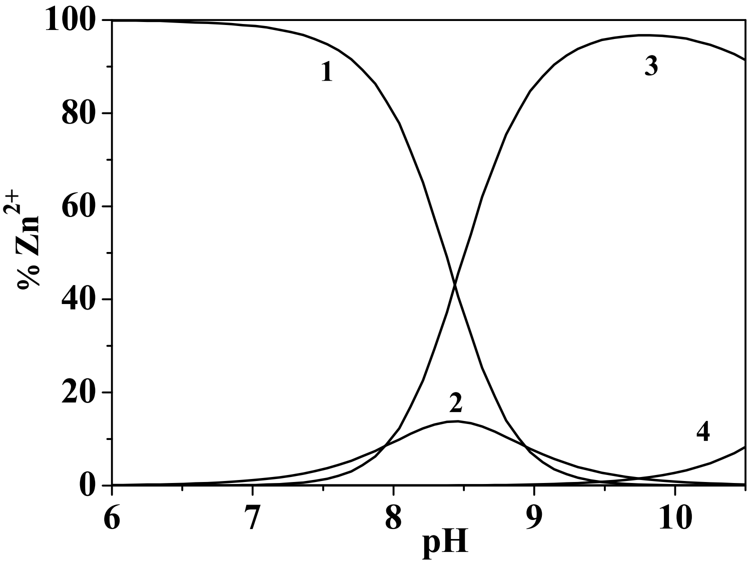

Figure S10. Distribution diagram of Zn^{2+} ($c_{Zn^{2+}}$ = 2.0·10^{-3} mol L^{-1}) species in absence of ionic medium and T = 298.15 K. Species: 1. free Zn^{2+}; 2. $Zn(OH)^{+}$; 3. $Zn(OH)_2{}^0{}_{(aq)}$; 4. $Zn(OH)_3{}^{-}$.

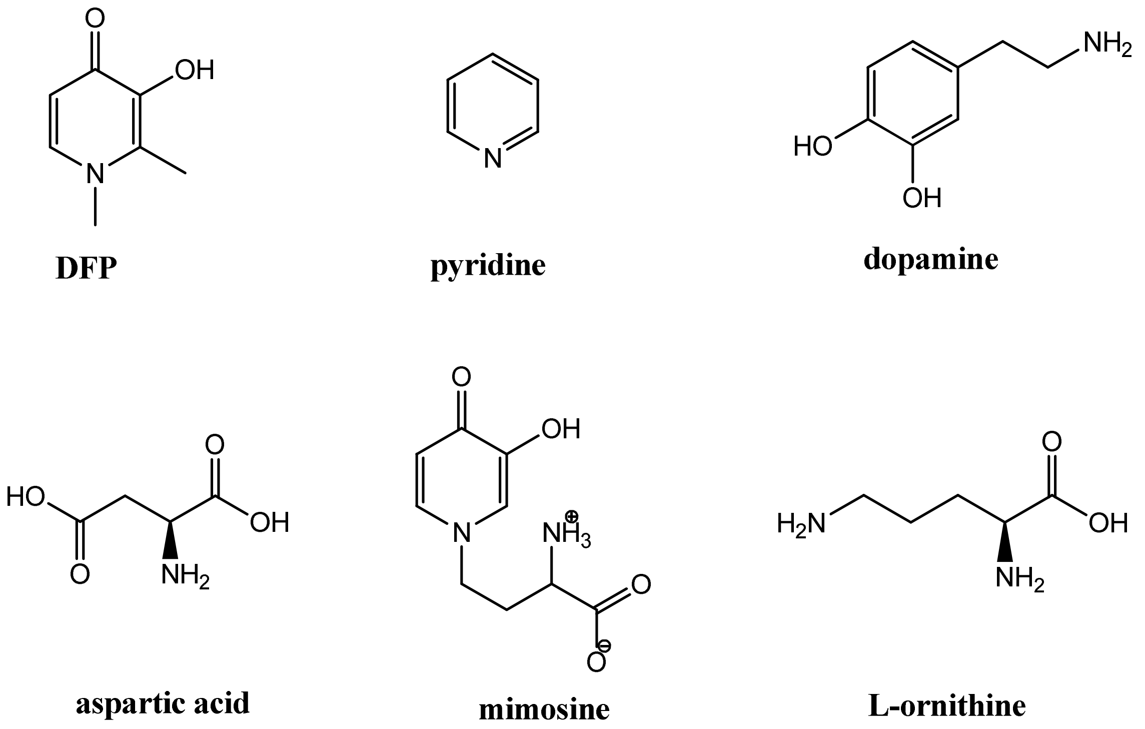

Figure S11. Molecular structures of products with similar structures and functional groups than 3-hydroxy-4-pyridinone ligands.
